# AXL-specific single domain antibodies show diagnostic potential and anti-tumor activity in Acute Myeloid Leukemia

**DOI:** 10.7150/thno.91456

**Published:** 2024-04-15

**Authors:** Niels Vandewalle, Hatice Satilmis, Emma Verheye, Rong Fan, Yanmeng Wang, Timo W.M. De Groof, Jessica Bridoux, Tessa Kerre, Nathan De Beule, Ann De Becker, Elke De Bruyne, Eline Menu, Karin Vanderkerken, Karine Breckpot, Nick Devoogdt, Kim De Veirman

**Affiliations:** 1 Translational Oncology Research Center (TORC), team Hematology and Immunology (HEIM), Vrije Universiteit Brussel, Brussels, Belgium.; 2 Laboratory of Dendritic Cell Biology and Cancer Immunotherapy, VIB Center for Inflammation Research, Brussels, Belgium.; 3 Brussels Center of Immunology, Vrije Universiteit Brussel, Brussels, Belgium.; 4 Laboratory of Molecular Imaging and Therapy (MITH), Vrije Universiteit Brussel, Brussels, Belgium.; 5 Department of Hematology, Ghent University Hospital, Ghent, Belgium.; 6 Translational Oncology Research Center (TORC), team Hematology and Immunology (HEIM), Universitair Ziekenhuis Brussel, Brussels, Belgium.; 7 Translational Oncology Research Center (TORC), Laboratory for Molecular and Cellular Therapy (LMCT), Vrije Universiteit Brussel, Brussels, Belgium.

**Keywords:** single domain antibodies, AXL, nuclear imaging, therapy, acute myeloid leukemia

## Abstract

**Rationale:** AXL expression has been identified as a prognostic factor in acute myeloid leukemia (AML) and is detectable in approximately 50% of AML patients. In this study, we developed AXL-specific single domain antibodies (sdAbs), cross-reactive for both mouse and human AXL protein, to non-invasively image and treat AXL-expressing cancer cells.

**Methods:** AXL-specific sdAbs were induced by immunizing an alpaca with mouse and human AXL proteins. SdAbs were characterized using ELISA, flow cytometry, surface plasmon resonance and the AlphaFold2 software. A lead compound was selected and labeled with ^99m^Tc for evaluation as a diagnostic tool in mouse models of human (THP-1 cells) or mouse (C1498 cells) AML using SPECT/CT imaging. For therapeutic purposes, the lead compound was fused to a mouse IgG2a-Fc tail and *in vitro* functionality tests were performed including viability, apoptosis and proliferation assays in human AML cell lines and primary patient samples. Using these *in vitro* models, its anti-tumor effect was evaluated as a single agent, and in combination with standard of care agents venetoclax or cytarabine.

**Results:** Based on its cell binding potential, cross-reactivity, nanomolar affinity and GAS6/AXL blocking capacity, we selected sdAb20 for further evaluation. Using SPECT/CT imaging, we observed tumor uptake of ^99m^Tc-sdAb20 in mice with AXL-positive THP-1 or C1498 tumors. In THP-1 xenografts, an optimized protocol using pre-injection of cold sdAb20-Fc was required to maximize the tumor-to-background signal. Besides its diagnostic value, we observed a significant reduction in tumor cell proliferation and viability using sdAb20-Fc *in vitro*. Moreover, combining sdAb20-Fc and cytarabine synergistically induced apoptosis in human AML cell lines, while these effects were less clear when combined with venetoclax.

**Conclusions:** Because of their diagnostic potential, sdAbs could be used to screen patients eligible for AXL-targeted therapy and to follow-up AXL expression during treatment and disease progression. When fused to an Fc-domain, sdAbs acquire additional therapeutic properties that can lead to a multidrug approach for the treatment of AXL-positive cancer patients.

## Introduction

Acute myeloid leukemia (AML) is a hematological malignancy characterized by the abnormal proliferation of immature myeloid cells and bone marrow failure 1. The backbone therapy for AML patients consists of a combination of daunorubicin and cytarabine, also called the “7+3 induction regimen” 2, 3. Unfortunately, because of increased treatment-related toxicity and unfavorable biological characteristics when compared to younger patients, the treatment of elderly AML patients (> 60 years) is more challenging. Therefore, their prognosis is poor with a 5-year overall survival of less than 20% 4. Since AML is a highly heterogeneous disease, both clinically and genetically, there is an increased demand for patient-tailored approaches to diagnose and treat AML patients 5.

Receptor tyrosine kinase AXL, derived from the Greek word 'anexelecto' (uncontrollable), has been identified as a prognostic factor and therapeutic target in AML patients 6. Binding of the ligand growth arrest-specific factor 6 (GAS6) to the AXL receptor was found to regulate various processes in cancer pathogenesis, including proliferation, survival, migration, stemness and chemoresistance 7, 8. In normal tissues, the AXL receptor can be found on the surface of, among others, bone marrow stroma and myeloid cells, where activated AXL will efficiently dispose apoptotic material and reduce TLR-dependent inflammatory responses and natural killer cell activity. In cancer, AXL is expressed on tumor cells as well as adjacent immune cells (e.g., dendritic cells, macrophages), and is often considered as an inhibitor of the innate immune response 9.

Several AXL targeting compounds have been developed, including small molecule inhibitors (e.g., bemcentinib, dubermatinib) and human anti-AXL monoclonal antibodies (mAbs) (e.g., tilvestamab) 8. Bemcentinib (also known as BGB324 or R428) is by far the most advanced anti-AXL therapeutic agent and was given FDA fast-track designation for the treatment of elderly patients with relapsed/refractory AML (NCT02488408). Since AXL is detectable in approximately 50% of the AML patients and appears to be highly predictive for response to AXL-targeted therapies (like bemcentinib) 6, monitoring AXL expression in cancer patients is highly recommended. While bone marrow biopsies are typically taken at diagnosis, repeated invasive biopsy procedures are considered painful and uncomfortable 10. Moreover, a single-site biopsy may not provide a representative profile of the tumor heterogeneity as it is spatially and temporally constrained, and necessitates the development of a non-invasive tool to follow-up AXL expression during and/or after therapy 8, 11.

In this study, we developed alpaca-derived, small sized single domain antibodies (sdAbs, 12-15 kDa), cross-reactive for mouse and human AXL protein, and evaluated their potential in a diagnostic and therapeutic context in AML. SdAbs present unique advantages over conventional mAbs due to their small size, allowing them to target and bind obstructed antigen binding sites and penetrate tissues effectively. Moreover, the fast blood clearance of radiolabeled sdAbs allows an early acquisition of high-quality PET images and fast diagnosis of patients, with low radiation burden. Additionally, sdAbs exhibit remarkable stability, solubility, and resistance to proteases, making them promising candidates for drug development with lower immunogenicity and simplified production processes compared to conventional mAbs 12, 13.

Several anti-AXL sdAbs were characterized using ELISA, flow cytometry, surface plasmon resonance and the AlphaFold2 prediction software. sdAb20 was selected as a lead compound and was evaluated as a diagnostic tool in mice bearing human (THP-1) or mouse (C1498) AML cells. For therapeutic purposes, sdAb20 was fused to a mouse IgG2a-Fc tail (sdAb20-Fc) and *in vitro* functionality tests were performed, including viability, apoptosis and proliferation assays using human AML cell lines and primary AML patient samples. The anti-tumor effect of sdAb20-Fc was evaluated in monotherapy, and in a dual combination with standard-of-care agents venetoclax or cytarabine, on human AML cell lines.

## Materials and Methods

### Animal studies

CB17-SCID and C57BL/6-Ly5.1 mice of 6-8 weeks old were purchased from Charles River (France and Germany, respectively). For imaging and biodistribution experiments, we used both the immunodeficient THP-1 xenograft model and the immunocompetent syngeneic C1498 mouse model. For the THP-1 xenografts, CB17-SCID mice were inoculated subcutaneously in the right flank with five million THP-1 cells that were diluted 1:1 with Matrigel (Corning, New York, USA). Tumor inoculation was performed under 2.5% isoflurane anaesthesia and subcutaneous tumor growth was allowed to a size of 250 ± 50 mm³. For the syngeneic C1498 mouse model, C57BL/6-Ly5.1 mice were inoculated intravenously with one million lentivirally transduced AXL^high^ C1498 cells under 2.5% isoflurane anaesthesia. C1498 mice were assessed daily for signs of leukemic disease (e.g., paralysis, weight loss). Prior to imaging, mice were anesthetized with 18.75 mg/kg of ketamine hydrochloride and 0.5 mg/kg of medetomidine hydrochloride. All animal experiments and housing conditions were approved by the Ethical Committee for Use of Laboratory Animals of the Vrije Universiteit Brussel and executed in accordance with the European guidelines for animal experimentation (License Agreement: LA 1230281, Files No.: 20-281-3, 21-281-2, 22-281-4, 23-281-1 and 23-281-4).

### Single domain antibody isolation and selection

Anti-AXL sdAbs were generated as previously described 14. In short, an alpaca (*Vicugna pacos*) was immunized with a mixture of 100 µg of recombinant mouse and 100 µg of recombinant human AXL proteins (R&D Systems, Oxon, UK; #154-AL, #854-AX) in Gerbu LQ 3000 adjuvant (GerbuBiotechnik GmbH, Heidelberg, Germany), at a rate of one injection per week for six weeks. Four days after the last immunization, 50 mL anti-coagulated blood was collected, lymphocytes were extracted with Leucosep (Greiner Bio-One, Kremsünster, Austria) by density centrifugation and total RNA was isolated and converted into cDNA by reverse transcription with oligo-dT primers. Gene fragments encoding the variable domain of camelid-specific heavy-chain-only antibodies were amplified via a two-step nested PCR and ligated into a pMECS phagemid vector. This vector was transformed into *E. coli* TG1 electrocompetent cells to generate a cloned sdAb-library of 10^8^ transformants. These were then phage displayed using M13K07 helper phages, and selected by biopanning, as described 15. After 3-4 consecutive rounds of *in vitro* selection on recombinant AXL protein-coated wells of Nunc Maxisorp flat bottom microtiter plates (Thermo Fisher scientific, Inc., Waltham, MA, USA), a total of 135 individual clones were selected and screened for binding on recombinant AXL proteins using enzyme-linked immunoassays (ELISA). In addition, their inserted DNA sequence was analyzed using the CLC Main Work Bench 8 software (Qiagen, Hilden, Germany), aligned according to the international ImMunoGeneTices (IMGT) database 16 and manually annotated.

### Bacterial production, purification and quality control of anti-AXL sdAbs

Based on their sequence, 118 clones were further selected and subdivided into 11 families and from the 135 original clones, 14 were selected and re-cloned into pHEN6 plasmids (to encode a C-terminal hexa-histidine (His6) tag) for expression of the monomeric version of the selected sdAbs. For production, the pHEN6-sdAb plasmids were transformed into an *E. coli* WK6 strain. The sdAbs were obtained via periplasmic expression and subsequently purified by immobilized metal affinity chromatography (IMAC) and size exclusion chromatography (SEC), as previously described 14. The concentration of the sdAbs was determined by spectrophotometry at 280 nm with Nanodrop^TM^ using the theoretically calculated extinction coefficient, based on the amino acid sequence. The purity of the sdAbs was confirmed by a 12% sodium dodecyl sulfate-polyacrylamide gel electrophoresis (SDS-PAGE) under reducing conditions, followed by Coomassie blue staining. Besides anti-AXL sdAbs, an irrelevant non-targeting control sdAb (referred to as R3B23), specific for 5T2 M-proteins 17, was used for *in vitro* and *in vivo* experiments. As a last step, the periplasmic proteins were screened for cross-reactive specificity against human and mouse AXL proteins using flow cytometry on the human HCT-116 and the murine CT-26 cell lines.

### Evaluation of affinity/kinetics using surface plasmon resonance

The affinity (K_D_-value) for both human and mouse AXL proteins of the purified sdAbs was determined using a Biacore-T200 device (GE Healthcare, Chicago, USA). The measurements were performed at 25°C using HEPES-Buffered Saline (HBS; 20 mM HEPES pH 7.4, 150 mM NaCl, 3.4 mM EDTA and 0.005% Tween 20) as running buffer. In short, recombinant AXL proteins were diluted and immobilized on a CM5 sensor chip Series S (GE-Healthcare) using amino coupling chemistry with 1-(3-(dimethylamino)propyl)-3-ethylcarbodi-imide (EDC) and N-hydroxy-succinimide (NHS). Remaining free EDC-NHS linkers were neutralized with 1 M ethanolamine-HCl pH 8.5. Seven serial dilutions were made of the purified sdAbs, after which the association (240 s) and dissociation (300 s) of each dilution to the immobilized recombinant AXL proteins were analyzed at a flow rate of 10 µL/min. After each cycle, the chip was regenerated twice for 30 s using 100 mM Glycine HCl pH 2.0 to completely remove any leftover sdAb. The response units from the flow channel on which recombinant AXL protein was immobilized were corrected by subtraction of the response units measured on a flow channel without protein. Additionally, the signal from the blank (HBS only) was subtracted from each measurement. The rate kinetic constants were determined by a mathematical fitting of a 1:1 binding model using the Biacore Evaluation software (GE Healthcare), and the k_on_/k_off_ ratio was used to determine the equilibrium dissociation constant (K_D_).

### ^99m^Tc-labeling of sdAbs, pinhole SPECT/CT imaging and image analysis

Radiolabeling of sdAb20 using ^99m^Tc-tricarbonyl chemistry was performed as previously described 18. In short, [^99m^Tc(H_2_O)_3_(CO)_3_]^+^ was linked to the C-terminal His_6_-tag of the sdAb using the Isolink® labeling kit (Mallinckrodt Medical BV, Petten, The Netherlands). Next, unbound [^99m^Tc(H_2_O)_3_(CO)_3_]^+^ was separated from the ^99m^Tc-sdAb complex via gel filtration on a pre-equilibrated NAP-5 SEC column (Sephadex, GE Healthcare, Machelen, Belgium), followed by filtration through a 0.22 µm filter (Millex, Millipore, Haren, Belgium). Labeling efficiency and purity (> 95%) were assessed using instant thin-layer chromatography (iTLC-SG, Pall Corporation, Hoegaarden, Belgium). Mice bearing subcutaneous THP-1 tumors and C1498 mice reaching humane endpoints were intravenously injected with 100 µL ^99m^Tc-labeled anti-AXL sdAb20 (5 µg) or the irrelevant ^99m^Tc-labeled control sdAb R3B23 with on average 37.9 ± 18.3 MBq of injected activity. For the THP-1 xenograft model, mice were pre-inoculated with unlabeled sdAb20-Fc (90 µg) construct for a variety of timepoints (0 h/co-injection, 2, 4, 6, 8, 24, 48 and 72 h) to reduce aspecific uptake of ^99m^Tc-labeled sdAb20. Pre-inoculation with sdAb20-Fc was not performed for imaging in the C1498 syngeneic AML model. One hour post-injection, pinhole SPECT-micro-CT imaging was performed using the MILabs VECTor/CT. SPECT images were generated using a rat SPECT-collimator (1.5 mm 75-pinhole general purpose collimator), in spiral mode, with six bed positions for whole-body imaging, 150 s per position. The CT-scan was set to 60 kV and 615 µA with a resolution of 80 µm. The total body scan duration was 15 min for SPECT and 139 s for CT. Images were reconstructed with 0.4 mm voxels with two subsets and seven iterations, without post-reconstruction filter. Images were analyzed using Medical Image Data Examiner (AMIDE) software and maximum intensity projections were generated using OsiriX Lite software (Pixmeo SARL, CH1233 Bernex, Switzerland).

### DTPA-conjugation and^ 111^In-labeling of Fc-conjugated sdAb constructs

All buffers were prepared metal free. SdAb-Fc constructs were buffer exchanged to 0.5 M Na_2_CO_3_ (pH 8.9-9.5 for R3B23-Fc; pH 9.5-9.9 for sdAb20-Fc) and concentrated to ± 4 mg/mL using Vivaspin 2 (MWCO 5000) (Sartorius), according to manufacturer's guidelines. For DTPA-conjugation, a 100-fold molar excess of NCS-DTPA (C_22_H_28_N_4_O_10_S·3HCL; S-2-(4-Isothiocyanatobenzyl)-diethylenetriamine pentaacetic acid; Macrocyclics) was added to the sdAb-Fc constructs and incubated for 2.5 h at room temperature with gentle shaking. The conjugated sdAb-Fc-DTPA complex was purified from excess NCS-DTPA via SEC (conjugation yield ~ 30%) on a Superdex 75 Increase 10/300GL column (GE Healthcare) with elution in 0.1 M NH_4_OAc (pH 7.0). The concentration of the conjugate was determined using a NanoDrop^TM^ 2000 spectrophotometer (Thermo Scientific), based on the new molecular weight and extinction coefficient of the sdAb-Fc-DTPA complexes.

For Indium-111 (^111^In)-labeling, 175-200 MBq of [^111^In]InCl_3_ (Curium Pharma, Petten, The Netherlands) was added to 75 µg DTPA-conjugated sdAb and 0.25 M NaOAc (pH 4.7) buffer, in a final volume of 500 µL. The reaction mixture was incubated for 30 min at room temperature, after which the radiotracer was purified via gel-filtration with a pre-equilibrated (5 mg/mL Vitamin C in 0.99% NaCl) NAP-5 column (GE Healthcare, Diegem, Belgium), according to manufacturer's protocol (decay-corrected radiochemical yield > 60%). Radiochemical purity (> 90%) was confirmed through iTLC-SG (Pall Corporation, Hoegaarden, Belgium), with 0.1 M sodium citrate (pH 5.0) as mobile phase, and via SEC on a Superdex 75 Increase 10/300 GL column (Cytiva) in 2x PBS at a flow rate of 0.8 mL/min. In addition, a radio-SDS-PAGE was performed, followed by Coomassie Blue staining, using a Novex^TM^ WedgeWell^TM^ 16% Tris-Glycerine Gel (Invitrogen) and a 1x Tris/Glycerine/SDS running buffer (Bio-Rad). The PageRuler™ Prestained Protein Ladder (Thermo Scientific^TM^) was used as a reference. Images were recorded with an Amersham Imager 680 and radioactive signals were detected with an autoradiograph Typhoon FLA 7000 Laser Scanner.

To confirm functionality, radio-ELISA was performed by coating Lockwell C8 Maxisorp plates (Thermo Scientific) with recombinant mouse antigen (R&D Systems, Oxon, UK; #854-AX). A serial dilution (300-11.1 nM) of the [^111^In]In-DTPA-sdAb-Fc constructs were added to the pre-coated plates, in the presence of PBS + 1% FBS. Plates were washed three times and binding was determined by assessing the radioactive counts with a γ-counter (Cobra Inspector 5003, Canberra, Packard, Illinois, IL, USA). Specific binding (K_D_) was calculated as the difference between total and non-specific binding.

Prior to *in vivo* injection, the radiolabeled tracers were passed through a 0.22 µm PVDF low protein binder filter (Millipore, Merck, Darmstadt, Germany), after which stability at room temperature was confirmed up to 72 h by SEC on a Superdex 75 increase 5/150 GL column in 2x PBS at a flow rate of 0.45 mL/min. Mice bearing subcutaneous THP-1 tumors were intravenously injected with 100 µL ^111^In-labeled anti-AXL sdAb20-Fc (5 µg) or the irrelevant ^111^In-labeled control sdAb R3B23 with on average 9.76 ± 2.34 MBq of injected activity.

### *Ex vivo* biodistribution

After imaging, mice were sacrificed for an *ex vivo* biodistribution study. Several organs, tissues and tumors were isolated to evaluate the specific uptake of the tracer using a γ-counter (Cobra Inspector 5003, Canberra, Packard, Illinois, IL, USA). The tissue/organ/tumor uptake was corrected for decay and calculated as the percentage of injected activity per gram tissue (%IA/g). For the C1498 mouse model, a single cell suspension was made of various organs known for C1498 AML infiltration, including blood, bone marrow (BM), liver, lungs, spleen, and ovary. Therefore, organs were crushed and cell suspensions were filtered through a 70 µm nylon filter to obtain a single cell suspension. Erythrocytes were lysed using red blood cell lysis buffer (0.16 M NH_4_Cl, 0.17 M Tris, pH 7.2). Finally, cells were washed, centrifuged, counted (Countess™ Automated Cell Counter, Invitrogen) and prepared for flow cytometry staining and analysis.

### Drugs and reagents

For *in vitro* experiments, anti-AXL sdAb20 and control sdAb R3B23 were reformatted into an Fc-construct (mIgG2a) and purchased from ImmunoPrecise Antibodies (Utrecht, Netherlands). The constructs were dissolved in PBS at a stock concentration of 3.71 mg/mL (sdAb20-Fc) and 3.11 mg/mL (R3B23-Fc). Three additional compounds were used during *in vitro* experiments: R428, cytarabine, and venetoclax. The small molecule AXL inhibitor R428 (BGB324, Bemcentinib) was obtained from SelleckChem (Munich, Germany) and dissolved in dimethylsulfoxide (DMSO; Sigma-Aldrich, St Louis, MO, USA) at a stock concentration of 10 mM. The chemotherapeutic agent cytarabine was purchased from Sigma (#147-94-4) and dissolved in sterile water at a stock concentration of 10 mM. Lastly, the BCL-2 inhibitor venetoclax (ABT-199) was obtained from SelleckChem (#1257044-40-8) and dissolved in DMSO at a stock concentration of 20 mM. All compounds were stored at -20°C.

### Cell viability and apoptosis assay

50,000 cells were seeded in 500 µL serum-supplemented medium in a 24-well plate and were treated with R428, R3B23-Fc, or sdAb20-Fc constructs as a single treatment or in combination with cytarabine or venetoclax at increasing concentrations for 72 h. We treated the cells with 200 µg/mL R3B23-Fc construct and 10, 100, or 200 µg/mL of the sdAb20-Fc construct or R428. We selected the final working concentrations for cytarabine of 250 nM, 500 nM, and 1000 nM for the MOLM-13 cell line and 1, 2, and 4 µM for the THP-1 cell line. Lastly, final working concentrations for venetoclax were 100, 500, and 1000 nM for the MOLM-13 cell line and 15, 30, and 60 nM for the THP-1 cell line. Used concentrations were determined based on titration experiments, resulting in low, medium, and high effects observed in the cell lines. Cell viability was analyzed using the CellTiter-Glo Luminescent Cell Viability Assay (Promega, Madison, WI, USA) and normalized to the untreated control sample. The remaining cell suspension was used to evaluate cell death (apoptosis) via AnnexinV-APC/7-AAD staining (BD Biosciences) and flow cytometry using the BD Accuri^TM^ C6 Plus and FACSCanto flow cytometers (BD Biosciences). Data were processed using BD FACSDiva^TM^ (BD Biosciences) and FlowJo 10.9® software (Tree Star; Inc., Ashland, OR, USA).

### BrdU incorporation assay and cell cycle analysis

AML cell proliferation was assessed by the incorporation of 5-Bromo-2-deoxyUridine (BrdU) during DNA replication. Cells were cultured at a concentration of 500,000 cells/mL and treated with 200 µg/mL R3B23-Fc and increasing concentrations of sdAb20-Fc (10, 100, 200 µg/mL) for 48 h. Four hours prior to sample collection, 1 mg/mL BrdU (Sigma, #B5002) was added to the medium. Samples were washed and fixated/incubated for 10 min at 4°C with a 4% paraformaldehyde solution, followed by overnight incubation in PBS + 0.2% Tween (Sigma-Aldrich) at room temperature. Cells were then washed twice, stained for 30 min with 2 M HCl at room temperature and washed with FACS flow and a mixture of PBS + 0.5% Triton-X (Sigma-Aldrich) + 10% FCS (PTF), respectively. Thereafter, cells were stained with 3 µL of FITC-conjugated Anti-BrdU-Fluorescein Antibody (Sigma-Aldrich, #1120269001) in 50 µL of PBS + 0.5% Triton-X + 10% FCS and incubated in the dark for 30 min. After washing, cells were incubated with a Cell Cycle Analysis (CCA) staining solution (0.1% Triton-X + 1 mg/mL Na₃C₆H₅O₇ + 50 µg/mL Propidium Iodide + 100 µg/mL RNase A). The percentage of BrdU+ cells was detected via flow cytometry using the FACSCanto and LSRFortessa flow cytometers (BD Biosciences) and gathered data were processed using BD FACSDiva^TM^ (BD Biosciences) and FlowJo 10.9® software (Tree Star; Inc., Ashland, OR, USA).

### Isolation of whole mononuclear cells from bone marrow samples

Bone marrow samples were collected as part of routine diagnostic or evaluation procedures after patients' signed informed consent and in accordance with the Declaration of Helsinki and an institutional research board approval from the University Hospital of Brussels (BUN: 1432021000659, Table S3). Mononuclear cells were isolated using density gradient centrifugation with Lymphoprep™ (STEMCELL™ technologies, Grenoble, France) and stored in liquid nitrogen until further needed. Upon thawing, cells were first resuspended in Roswell Park Memorial Institute (RMPI)-1640 medium (Gibco; Thermo Fisher Scientific), supplemented with 20% fetal calf serum (FCS) (PAN Biotech, Aidenbach, Bayern, Germany), 100 U/mL penicillin, 100 µg/mL streptomycin, 2 mM L-glutamine (Lonza, Basel, Switzerland), 100 µg/mL DNase I (Merck, Belgium) and 10 mM MgCl_2_. After 30 min of incubation and 10 min of centrifugation at 400g, the cells were washed twice with RPMI-1640 medium with 20% FCS and then counted with Trypan Blue staining to determine the number of dead cells.

### Statistical analysis

Statistical analyses were performed with GraphPad Prism 8.01 software (GraphPad Software Inc., La Jolla, CA, USA). All data are represented as mean ± standard deviation (SD). p-values were calculated using a one-sided Mann-Whitney U t-test (two groups) or a one-way ANOVA (three or more groups). p ≤ 0.05 (*), p ≤ 0.01 (**), p ≤ 0.001 (***) and p ≤ 0.0001 (****) were considered statistically significant. Normality was confirmed using the Shapiro-Wilk and Kolmogorov-Smirnov tests.

## Results

### Development and *in vitro* characterization of cross-reactive AXL-targeting single domain antibodies

**Figure 1:**
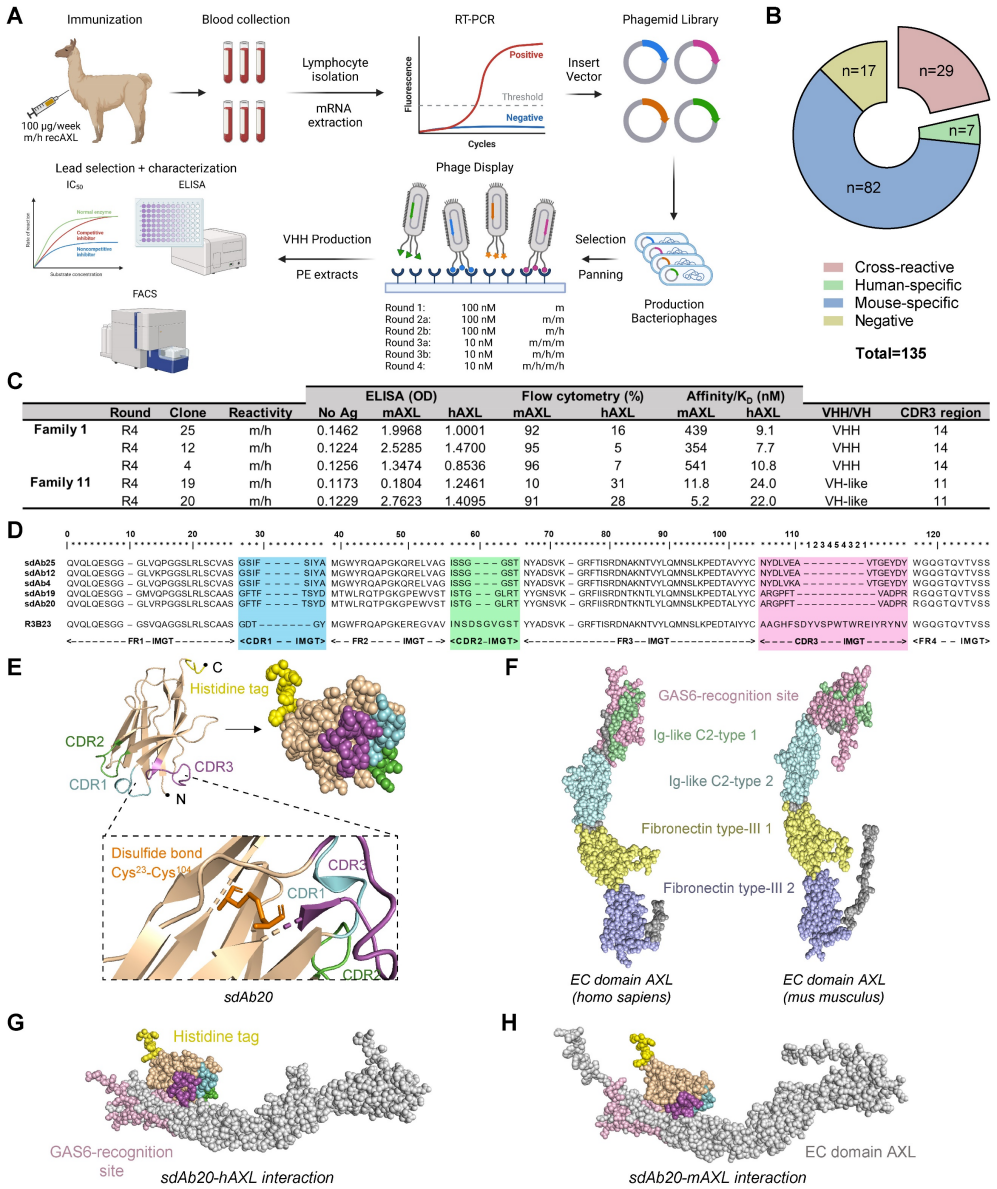
** Generation and initial characterization of AXL-specific single domain antibodies. (A)** Schematic illustration of alpaca immunization strategy, sdAb library construction and phage display selection of AXL-specific sdAbs. mRNA from blood lymphocytes served as a template for sdAb-specific PCR reactions, and resultant purified fragments were cloned into phagemids. Subsequently, antigen-specific phages were identified from this phagemid library using ELISA, flow cytometry and surface plasmon resonance. Figure created with BioRender.com.** (B)** Initial screening of 135 selected clones allowed for further classification into negative, mouse-specific, human-specific and cross-reactive. **(C)** Based on their characteristics, five cross-reactive sdAbs were selected for further investigation. The table shows a summary of their most important features. **(D)** Amino acid sequences of the selected anti-AXL sdAbs (numbering accor-ding to IMGT) 14. The CDR1, CDR2 and CDR3 regions are highlighted in cyan, green and magenta, respectively.** (E-F)** Structural analy-sis of the selected sdAb20 and the extracellular domain of both murine and human AXL using Alphafold2 and Pymol software. **(G-H)** Alpha-Fold2 prediction of the interaction between sdAb20 and AXL. CDR = complementarity-determining regions; EC = extracellular; FR = frame-work region.

AXL has been shown to be overexpressed in different tumor types, including AML 6, 7, 19. Since this protein is not only expressed by tumor cells, but also by various immune cells (e.g., macrophages, dendritic cells) 7, 20, evaluation in preclinical immunocompetent models is indispensable. To aid clinical translation, we immunized an alpaca with both mouse and human AXL proteins (homology ~88%, determined using the Ensembl Database), followed by four rounds of biopanning of the resulting phage-displayed immune sdAb library (Figure 1A). Upon sequencing, 118 unique anti-AXL sdAbs (subgrouped in 11 families based on CDR3 sequence similarities) were identified and sdAbs were classified as either mouse specific (n=82), human specific (n=7) or cross-reactive (n=29) (Figure 1B-C). To confirm this, periplasmatic extracts were screened by ELISA for binding to recombinant mouse and human AXL proteins (Figure 1C). Next, we evaluated the cell binding potential of sdAbs to AXL^+^ cancer cells (human HCT-116, murine CT26) using flow cytometry and sdAb affinity was determined using surface plasmon resonance (Figure 1C, Figure S1A,B). Based on all *in vitro* characteristics, five cross-reactive sdAbs were selected for amino acid sequence alignment (according to IMGT) (Figure 1D). From these five sdAbs, sdAb20 was selected as the lead compound for further *in vitro* and *in vivo* evaluation (Figure 1C-D), based on its properties, including low-nM affinity binding, cross-reactivity for mouse and human AXL, thermal stability (> 50°C; Figure S1C) and the ability to specifically block the interaction between GAS6 and AXL. Using AlphaFold2 and Pymol software, the 3D structure of sdAb20 (Figure 1E) and the extracellular domain of mouse and human AXL (Figure 1F), as well as the binding of sdAb20 (through its CDR1, CDR2 and CDR3 loops) to the AXL protein (Figure 1G-H, Figure S2A-D) was predicted. Interestingly, sdAb20 interacted with the GAS6 recognition region of the Ig-like C2-type 1 domain of AXL, possibly leading to blocking of the AXL-GAS6 interaction, and therefore suggesting that sdAb20 could also have a therapeutic effect.

### Binding potential of sdAb20 on human and murine AXL^+^ AML cell lines

**Figure 2:**
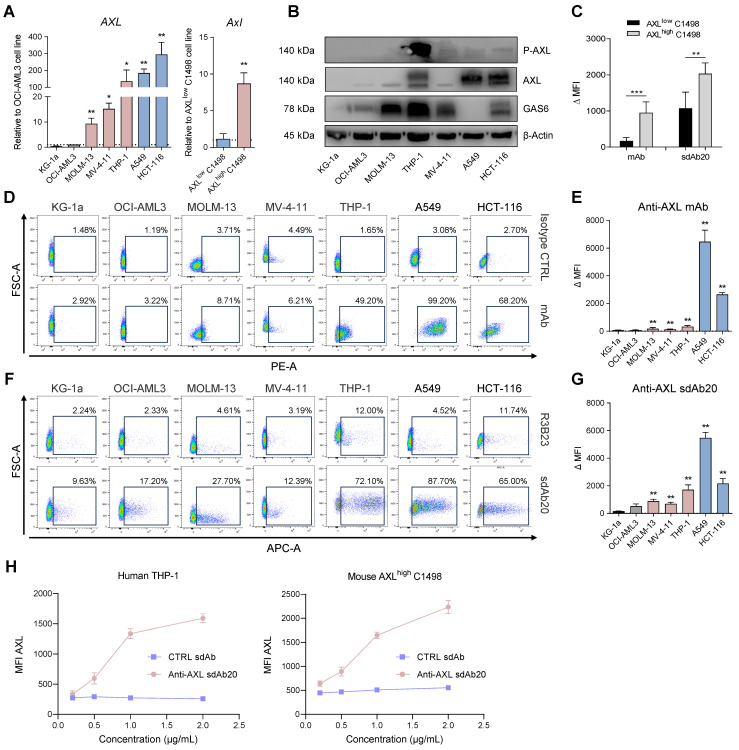
** sdAb20 can specifically distinguish AXL^high^ and AXL^low^ cancer cell lines. (A)** Human (AXL) and murine (Axl) gene expression levels of Axl were measured using qRT-PCR after RNA extraction from human AML (KG-1a, OCI-AML3, MOLM-13, MV-4-11 and THP-1), human non-small cell lung carcinoma (A549), human colorectal carcinoma (HCT-116) and murine AML (AXL^low^ C1498 and AXL^high^ C1498) cell lines. Human expression levels relative to OCI-AML3 cell line (n=5, ± SD). Murine expression levels relative to AXL^low^ C1498 cell line (n=5, ± SD). **(B)** Western blot analysis for protein expression of AXL, phosphorylated AXL (P-AXL) and GAS6 in extracts from several cancer cell lines (n=4).** (C)** Binding of sdAb20 to murine AML cell lines was assessed by flow cytometry and compared to an APC-coupled anti-AXL mAb. The anti-AXL mAb was compared to an isotype control and sdAb20 was compared to an irrelevant control sdAb (R3B23) (n=5, ± SD). **(D-E)** Flow cytometric assessment of AXL expression in several human cancer cell lines using an anti-AXL mAb and a corresponding isotype control (n=5, ± SD). **(F-G)** Flow cytometric analysis of the binding potential of sdAb20 to human cancer cell lines with a varying AXL expression. The irrelevant sdAb R3B23 served as a negative control (n=5, ± SD).** (H)** The specificity of the binding potential of sdAb20 to human (THP-1) and murine (C1498) AML cell lines was investigated by administering sdAb20 with increasing doses to two AML cell lines with a high AXL-expression (n=3, ± SD). p ≤ 0.05 (*), p ≤ 0.01 (**) and p ≤ 0.001 (***) were considered statistically significant. FSC = forward scatter, mAb = monoclonal antibody, MFI = median fluorescence intensity, ΔMFI = MFI (anti-AXL mAb) - MFI (isotype CTRL) or MFI (sdAb20) - MFI (CTRL sdAb).

Since AML is a commonly used model to evaluate AXL targeting therapeutics, we first assessed the expression of *AXL* in different human AML cell lines. Solid tumor cancer cells A549 (NSCLC) and HCT-116 (CRC) were included as positive controls. We observed a heterogeneous gene expression of *AXL* in AML cell lines, with the highest expression being detected in MV-4-11, MOLM-13, and THP-1 (Figure 2A). The highest protein expression of GAS6, AXL and phosphorylated (activated) AXL (P-AXL) was found in human THP-1 cells, while KG-1a cells were considered negative for AXL (Figure 2B). Since AXL expression is low in the murine C1498 AML cell line, we generated AXL-overexpressing C1498 cells (AXL^high^ C1498) by lentiviral transduction and positive selection (Figure 2A,C; Figure S3A-C). This AML model was further used to assess the murine cell binding potential and for *in vivo* evaluation.

Using flow cytometry, we compared the cell binding potential of sdAb20 to a commercially available mAb (Figure 2C-G). While the binding of the mAb to AXL^+^ AML cell lines resulted in a weak signal (Figure 2D-E), the MFI of the total population shifted significantly with sdAb20 compared to the control sdAb (R3B23) in AXL^+^ AML cells (Figure 2F-G). For solid tumor cell lines A549 and HCT-116, a clear AXL^+^ cell population could be distinguished using both the mAb and sdAb20. The binding of sdAb20 was also validated in the AXL^high^ C1498 model (Figure 2C) and we observed a dose-dependent increase in mean fluorescence intensity in the THP-1 and AXL^high^ C1498 cell lines (Figure 2H). These data illustrate the potential of sdAb20 to bind both murine and human AXL-expressing AML cells in a specific manner.

### Specific tumor uptake of ^99m^Tc-labeled sdAb20 in THP-1 xenografts

**Figure 3:**
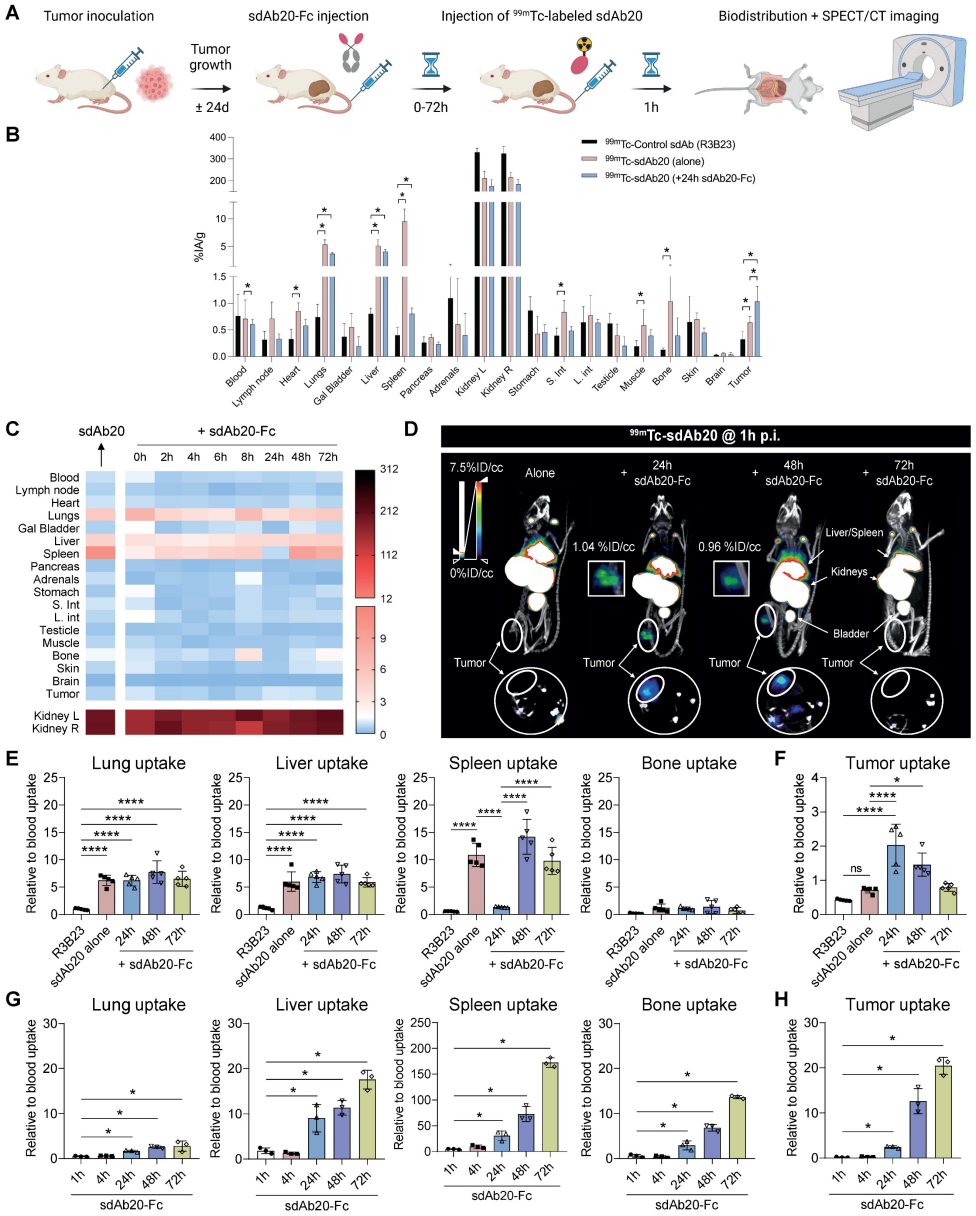
** sdAb20 can specifically recognize AXL-expressing cells in the human THP-1 AML xenograft mouse model. (A)** Schematic overview of an optimized protocol to investigate the biodistribution profile of sdAb20. 21-24 days post tumor-inoculation, mice will first receive a pre-determined dose of unlabeled, cold sdAb20-Fc, after which they are injected with radiolabeled (^99m^Tc) sdAb20 after 0-72 h. One hour later, mice will undergo SPECT/CT imaging and ex vivo biodistribution through organ collection. Figure created with BioRender.com. **(B)** The ex vivo biodistribution profile of ^99m^Tc-R3B23, ^99m^Tc-sdAb20 alone and ^99m^Tc-sdAb20 after pre-treatment with sdAb20-Fc for 24 h. Results are presented as mean %IA/g ± SD. **(C)** Heatmap of accumulation patterns of ^99m^Tc-sdAb20 in various organs at different timepoints upon pre-treatment with sdAb20-Fc. The colour key represents the mean value of tracer accumulation in organs (%IA/g, n=3 for each group, n=5 for 24 h group). **(D)** Reconstructed SPECT/CT images of THP-1 xenograft mice showing maximum intensity projection and transversal planes. One reconstructed image representing three individual mice. White dashed circles indicate THP-1 tumors. **(E)** Differences in lung, liver, spleen, bone and **(F)** tumor, relative to blood uptake. **(G)** Uptake of 111In-labeled sdAb20-Fc in lung, liver, spleen, bone and (H) tumor at different timepoints. Data were represented as organ-to-blood ratio. p ≤ 0.05 (*) and p ≤ 0.0001 (****) were considered statistically significant. IA = injected activity, ID/cc = injected dose per cubic centimeter.

To validate the potential of sdAb20 as a diagnostic tracer in AML, we performed biodistribution studies in naive mice (Figure S1D) and in the subcutaneous THP-1 xenograft model (Figure 3A). The biodistribution of ^99m^Tc-labeled sdAb20 in naive mice demonstrated an elevated uptake in especially lungs, liver and spleen (> 5 %IA/g) compared to radiolabeled R3B23 control sdAb (Figure S1D). Intravenous injection of ^99m^Tc-labeled sdAb20 in tumor-bearing mice and *ex vivo* gamma counting of the organs demonstrated a significant uptake of sdAb20 within the tumor compared to control sdAb R3B23 (Figure 3B; *p = 0.0040*). However, the subcutaneous tumor uptake remained undetectable by SPECT/CT imaging. While CB17-SCID mice are deficient in B- and T-cells, myeloid cells such as macrophages remain present and are known to also express the AXL protein 20. Therefore, sdAb20 was specifically taken up in organs like spleen, liver and lungs; resulting in a sink effect in these organs in tumor-bearing mice (Figure 3B-C). To reduce this background signal and increase tumor uptake, we further optimized our protocol using a pre-injection with cold (unlabeled) Fc-conjugated sdAb20. THP-1 xenografted mice were co-injected (0 h) or pre-treated with sdAb20-Fc for 2 h, 4 h, 6 h, 8 h, 24 h, 48 h and 72 h prior to ^99m^Tc-labeled sdAb20 administration and SPECT/CT imaging (Figure 3A). Only pre-treatment for 24 h with cold sdAb20-Fc resulted in a significant reduction of the signal in the spleen and a significant increase in tumor uptake (Figure 3B-F; *p = 0.0040*), which could be clearly visualized using SPECT/CT imaging (Figure 3D). However, the tumor uptake of sdAb20 reduced after pre-treatment with sdAb20-Fc for 48 h and 72 h (Figure 3B-F).

To further clarify the observed effect, we analyzed the biodistribution profile of radiolabeled sdAb20-Fc and control R3B23-Fc sdAb. As the His_6_-tag of sdAb20 has been replaced with a functional mIgG2a-Fc region, and given the lower pharmacokinetics of sdAb20-Fc (evaluations up to 72 h), labeling with ^99m^Tc (decay t_1/2_ = 6.0 hours) was no longer possible and had to be replaced by ^111^In (decay t_1/2_ = 2.8 days) instead. After DTPA-conjugation, sdAb20-Fc was radiolabeled with ^111^In (purity > 90%) and quality controls were performed, including radio-ELISA, radio-SDS-PAGE and a stability assay (radiochemical purity remained > 90% up to 72 h), to confirm the functionality of the compound (Figure S4A-E). Starting with a high uptake at 1 h post-injection in almost all organs, with spleen and blood having the highest %IA/g values (66.16 ± 6.51 and 9.80 ± 1.48 %IA/g, respectively), both sdAb-Fc constructs gradually decreased in circulation (Figure S5A-D). At 24 h post-injection, sdAb20-Fc primarily accumulated in the spleen (35.41 ± 3.73 %IA/g) and low uptake could be observed in liver (6.61 ± 0.69 %IA/g), bone (3.48 ± 0.52 %IA/g) and tumor (2.97 ± 0.35 %IA/g) (Figure 3G, Figure S5). At 48 h and 72 h, a significant increase in tumor-to-blood ratios could be observed for ^111^In-labeled sdAb20-Fc (12.99 ± 2.73 %IA/g and 23.86 ± 2.53 %IA/g, for 48 h and 72 h, respectively) (Figure 3H).

Taken together, these data illustrate that 24 h pre-treatment with sdAb20-Fc can reduce the uptake of ^99m^Tc-labeled sdAb20 in the spleen while significantly increasing tumor uptake. On the other hand, the elevated tumor accumulation levels of sdAb20-Fc at later time points probably resulted in a reduced imaging signal of ^99m^Tc-labeled sdAb20 using the 48 h and 72 h pre-treatment protocol.

### Specific uptake of ^99m^Tc-labeled sdAb20 in naive and immunocompetent AXL^high^ C1498 mice

**Figure 4:**
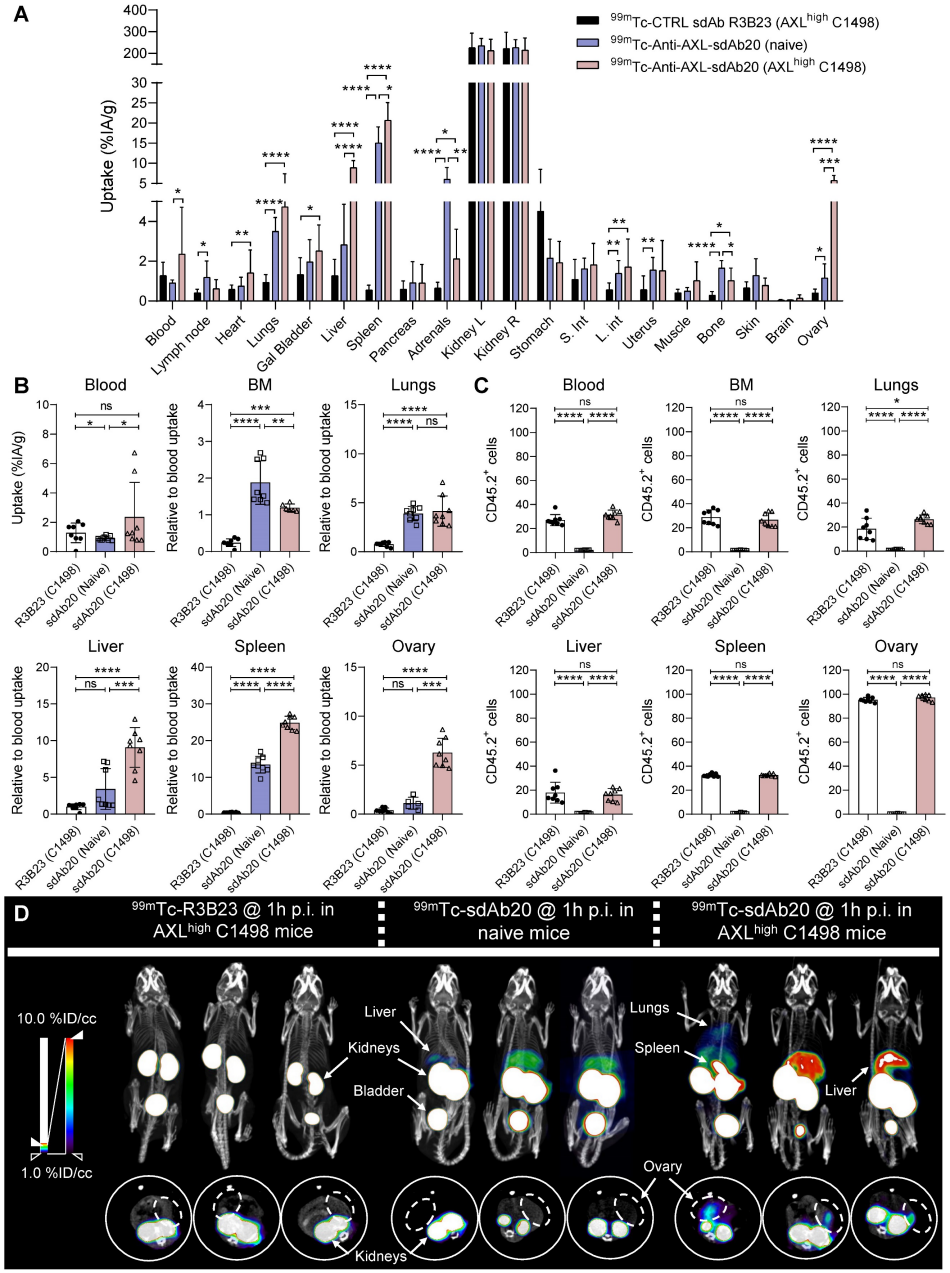
**^99m^Tc-sdAb20 can specifically target and visualize AXL-expressing cells in the syngeneic murine C1498 AML mouse model. (A)** Ex vivo γ-counting of isolated organs from naive and AXL^high^ C1498 mice. Organs were isolated 90 min post-injection of radiolabeled (^99m^Tc) sdAb20 or R3B23 control tracer (n=5). **(B)** Uptake of radiolabeled sdAb20 in various hematological organs of naive and AXL^high^ C1498 tumor-bearing immunocompetent mice. Data were represented as organ-to-blood ratio. **(C)** Flow cytometric analysis of end-stage tumorload (CD45.2+ tumor cells) of different organs (n=8, ± SD). **(D)** Reconstructed SPECT/CT images of naive and AXL^high^ C1498 mice, one hour post-injection of ^99m^Tc-labeled anti-AXL sdAb20 and R3B23 control sdAb (n=8). Images show maximum intensity projections and transversal planes; and are reconstructed with OsiriX software. Representative intensities (%IA/g) are shown next to the images. Dashed circles represent ovaries. %ID/cc = injected dose per cubic centimeter. p ≤ 0.05 (*), p ≤ 0.01 (**), p ≤ 0.001 (***) and p ≤ 0.0001 (****) were considered statistically significant.

Although the data in THP-1 xenografted mice already provided evidence for the use of sdAb20 as a diagnostic tool in AML, we aimed to further validate its potential using an immunocompetent syngeneic AML mouse model using the C1498 cell line. This murine AML cell line was isolated from C57BL/6 mice and is described to accumulate within the bone marrow, spleen, liver, lungs and ovaries upon intravenous injection 21. However, as AXL expression in C1498 cells is low, we lentivirally modified this cell line to overexpress the AXL protein (Figure S3A-C). Naive mice and tumor-bearing mice (21 days post tumor inoculation) were intravenously injected with ^99m^Tc-labeled sdAb20 or control sdAb R3B23, followed by SPECT/CT imaging after 1 h of biodistribution. In both naive and AXL^high^ C1498 mice, we observed a significant uptake of sdAb20 in lungs (*p < 0.0001*), liver (*p = 0.0035* and *p < 0.0001*), spleen (*p < 0.0001*), adrenals (*p < 0.0001* and *p = 0.0141*), bone marrow (*p < 0.0001* and *p = 0.0003*) and ovaries (*p = 0.0054* and *p < 0.0001*) compared to R3B23 (Figure 4A). Interestingly, AXL^high^ C1498 mice demonstrated a significant higher uptake in blood (*p = 0.0249*), spleen (*p < 0.0001*), liver (*p < 0.0001*), bone marrow (*p = 0.0007*) and ovary (*p = 0.0008*) compared to naive mice, in all organs that were found to be infiltrated with AXL^high^ C1498 (CD45.2^+^) cells (Figure 4B-C). Despite the accumulation of tumor cells within the lungs of AXL^high^ C1498 mice, no significant difference in *ex vivo* radioactivity could be found compared to naive mice (Figure 4B; *p = 0.3992*). Finally, SPECT/CT images illustrated a difference between the signal intensity of sdAb20 in AXL^high^ C1498 mice compared to naive mice, confirming the potential of sdAb20 as a diagnostic tool to identify AXL^+^ tumors (Figure 4D).

### Fc-coupled sdAb20 decreases cell viability and proliferation of human AXL positive AML cell lines *in vitro*

**Figure 5:**
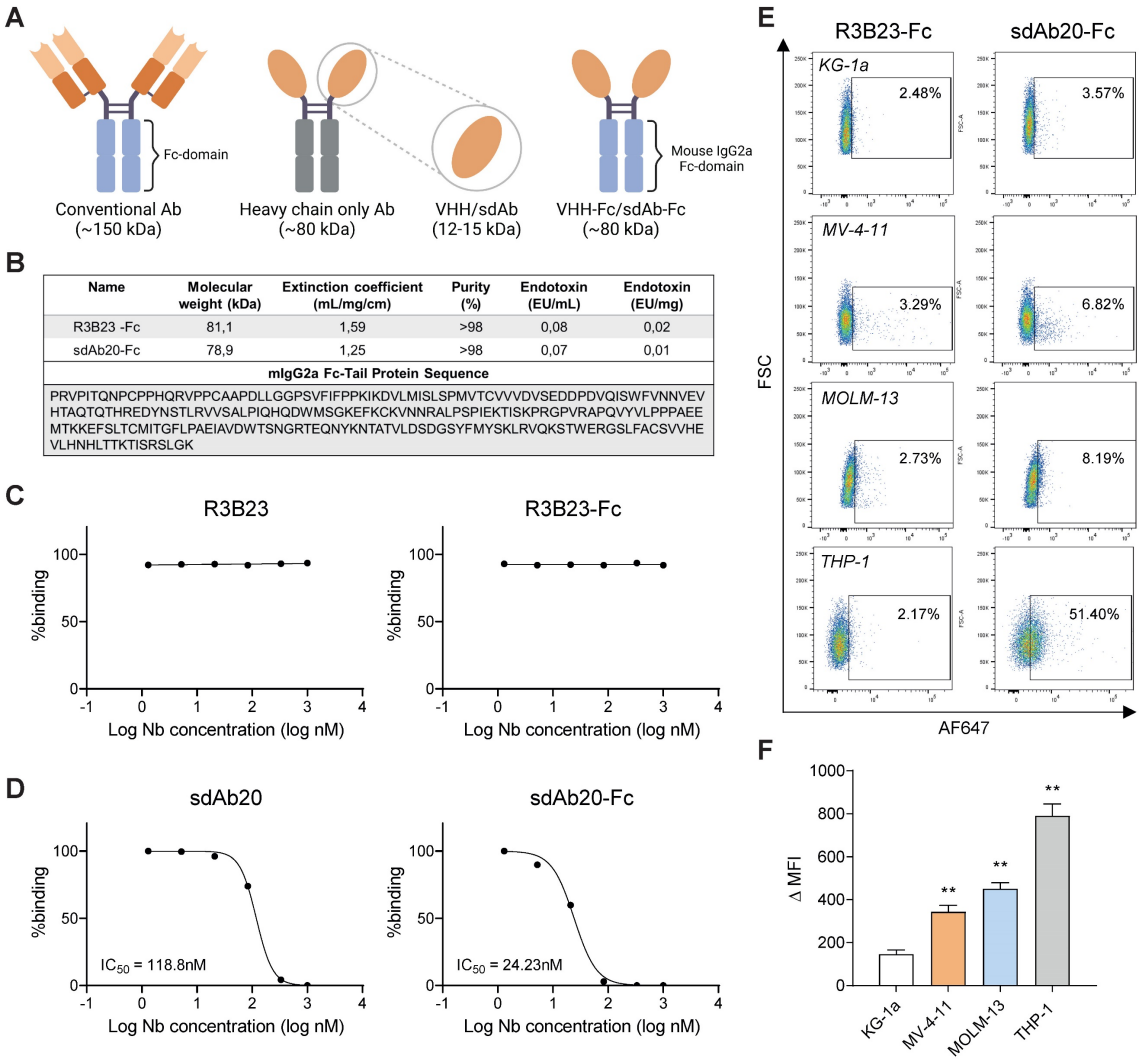
** sdAb20-Fc generation and characterization. (A)** Graphical representation of size difference between a conventional Ab, a heavy-chain only Ab, a sdAb and a sdAb-Fc construct. Figure created with BioRender.com. **(B)** Summarizing table of the most important characteristics of R3B23-Fc and sdAb20-Fc, including the mIgG2a-sequence that was used to generate these constructs. **(C-D)** Comparison of IC50 values, for blocking of hAXL and hGAS6, of sdAb20 and sdAb20-Fc as determined via a competition assay using surface plasmon resonance. **(E-F)** Flow cytometric analysis of the binding potential of sdAb20-Fc to human cancer cell lines with a varying AXL expression. The irrelevant sdAb R3B23-Fc served as a negative control (n=4, ± SD). p ≤ 0.01 (**) was considered statistically significant. MFI = median fluorescence intensity.

Since the binding of GAS6 to AXL has been found to promote cancer progression and sdAb20 was predicted to interact with the GAS6 recognition site, we further evaluated the GAS6/AXL blocking capacity of sdAb20 *in vitro* using a competition assay. Although sdAb20 could inhibit the binding of GAS6 to AXL (IC_50_ = 118.8 nM) (Figure 5C-D), we only observed a limited effect on AML cell viability or apoptosis (Figure S6A-B). To further improve the therapeutic capacity, sdAb20 was coupled to a mIgG2a Fc-domain, which has been described to offer superior anti-tumor activity to mIgG1 (Figure 5A-B) 22. SdAb20-Fc was able to bind AXL^+^ MV-4-11, MOLM-13 and THP-1 AML cells (Figure 5E-F), however the shift in fluorescence signal (MFI) was less clear compared to the monovalent sdAb20. Interestingly, the Fc-fused sdAb20 construct resulted in an increased GAS6/AXL blocking capacity with an IC_50_ of 24.23 nM (Figure 5C,D).

**Figure 6:**
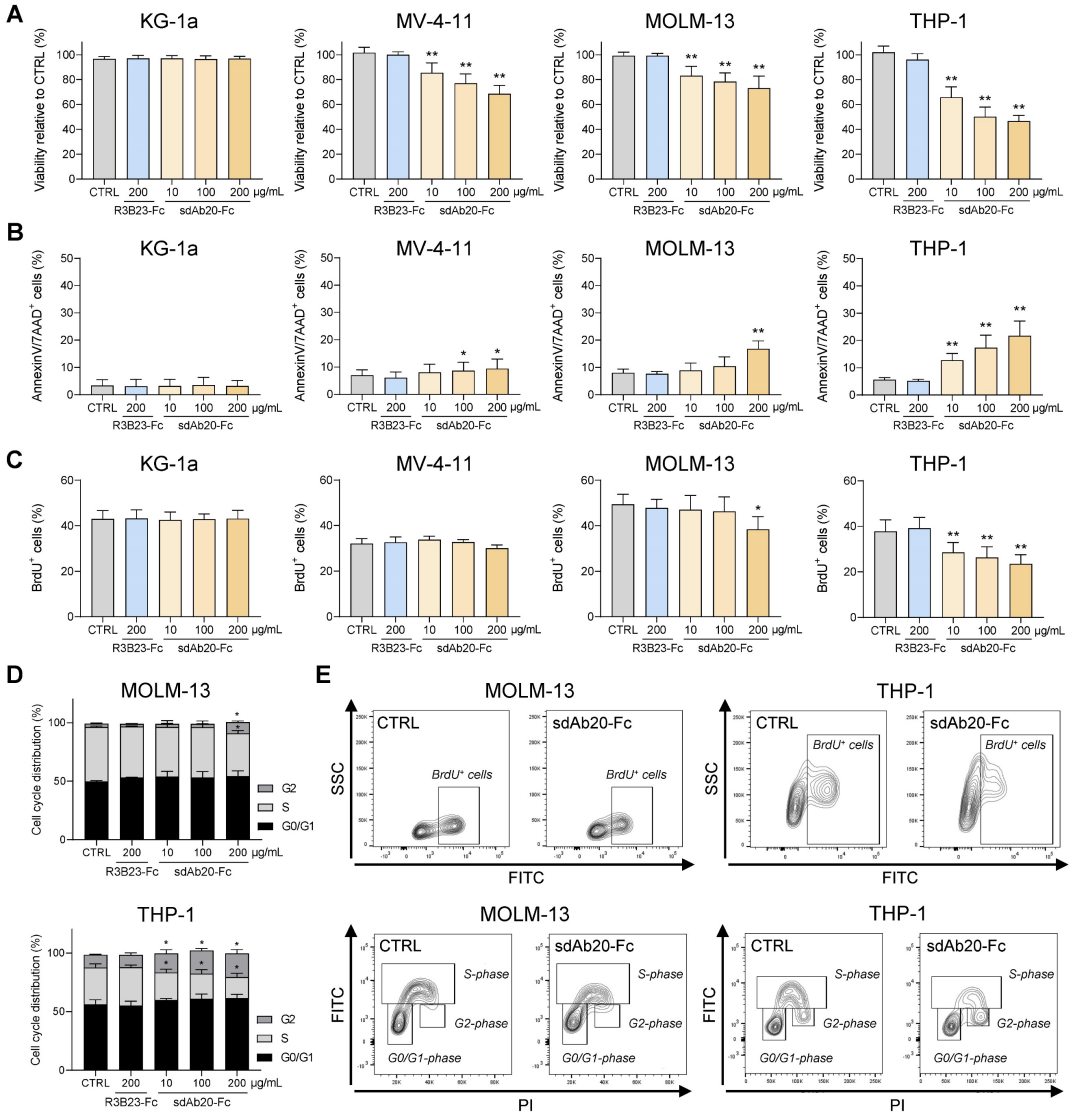
** sdAb20-Fc has promising anti-AML effects as a single agent. (A-B)** AML cell lines KG-1a, MV-4-11, MOLM-13 and THP-1 were treated with indicated concentrations of R3B23-Fc (200 μg/mL) or sdAb20-Fc (10, 100 and 200 μg/mL) for 72 h. The effect on cell viability (A) and apoptosis (B) was assessed via CellTiter-Glo assay and Annexin V/7-AAD staining, respectively (KG-1a: n=6; MV-4-11: n=6; MOLM-13: n=5; THP-1: n=6, ± SD). **(C)** MOLM-13 and THP-1 cells were treated with either R3B23-Fc or increasing concentrations of the sdAb20-Fc construct for 48 h. The number of proliferating cells was assessed using BrdU staining (THP-1: n=5; MOLM-13: n=3, ± SD). **(D)** Cell cycle analysis was performed on THP-1 and MOLM-13 cells using propidium iodide (PI) staining (THP-1: n=4; MOLM-13: n=3, ± SD). **(E)** Gating strategy used to determine the number of proliferating cells and to distinguish between the different cell cycle phases (G0/G1-phase, S-phase, G2-phase). p ≤ 0.05 (*) and p ≤ 0.01 (**) were considered statistically significant. CTRL = control.

Next, we assessed the therapeutic effect of sdAb20-Fc in AXL-positive AML cell lines MV-4-11, MOLM-13 and THP-1. KG-1a was included as negative control. Treatment of MV-4-11, MOLM-13 and THP-1 with sdAb20-Fc resulted in a dose-dependent decrease in AML cell viability (Figure 6A) and increased apoptosis (Figure 6B) compared to R3B23-Fc, with the most pronounced effect in the THP-1 cell line. SdAb20-Fc treatment did not affect the KG-1a cell line. To further clarify the underlying mechanism of the observed anti-AML effect, we evaluated the effect of sdAb20-Fc in GAS6^-^/AXL^+^ (A549) and GAS6^+^/AXL^+^ (HCT-116) solid tumor cell lines. We selected A549 and HCT-116 since no AML cell lines were available with these characteristics. Anti-AXL sdAb20-Fc therapy significantly reduced the viability of the GAS6^+^ HCT-116 cell line, while no effect could be observed for the GAS6^-^ A549 cell line (Figure S6C). Similar results were found on apoptosis (Figure S6D). These data indicate that the direct therapeutic effect of sdAb20-Fc on AXL^+^ tumors is mediated by blocking the interaction between GAS6 and AXL.

As a positive control, we also tested the FDA-approved small molecule inhibitor R428 (bemcentinib). We observed a significant reduction in cell viability in all tested AML cell lines, but in contrast to sdAb20-Fc also in the AXL-negative KG-1a cell line (Figure S7A-B). Moreover, a direct comparison of the anti-AML effects of R428 and sdAb20-Fc at different molar concentrations (ranging from 10 nM to 10 µM) clearly demonstrated that R428 reduced the viability of both the KG-1a and THP-1 cell lines, regardless of their AXL expression, in a dose-dependent manner (Figure S7C-D). Surprisingly, more potent killing effects could be observed in the AXL^-^ KG-1a cell line (IC_50_: 544.3 nM, Figure S7C) compared to the AXL^+^ THP-1 cell line (IC_50_: 1.2 µM, Figure S7D) after treatment with R428, indicating AXL-independent off-target effects. In contrast to these results, sdAb20-Fc did not have any effect on the viability of the AXL^-^ KG-1a cell line, emphasizing its AXL-specific capacities. For the THP-1 cell line, we were able to replicate our previous findings, with an IC_50_ of 2.3 µM for sdAb20-Fc (Figure S7D).

Lastly, we also tested the effect of sdAb20-Fc on AML cell proliferation. SdAb20-Fc treatment resulted in a significant decrease in the percentage of BrdU-positive AML cells compared to R3B23-Fc treatment (Figure 6C,E), which was associated with a decrease in the S-phase and a significant increase in the G2-phase (Figure 6D-E).

Taken together, these data illustrate the direct anti-proliferative and pro-apoptotic effects of the Fc-fused sdAb20 construct by blocking the interaction between AXL and GAS6 in AML.

### GAS6/AXL-targeting by sdAb20-Fc increases sensitivity towards standard-of-care agents in AML

The chemotherapeutic agent cytarabine and BCL-2 inhibitor venetoclax are commonly used drugs for the treatment of AML patients. In a first step, we evaluated the effect of both compounds on AXL expression in MOLM-13 and THP-1 cells using qRT-PCR and flow cytometry. Treatment with cytarabine for 48 h resulted in a significant increase of AXL expression in THP-1 and MOLM-13 cells, while this effect was absent with venetoclax (Figure S8A-D).

**Figure 7:**
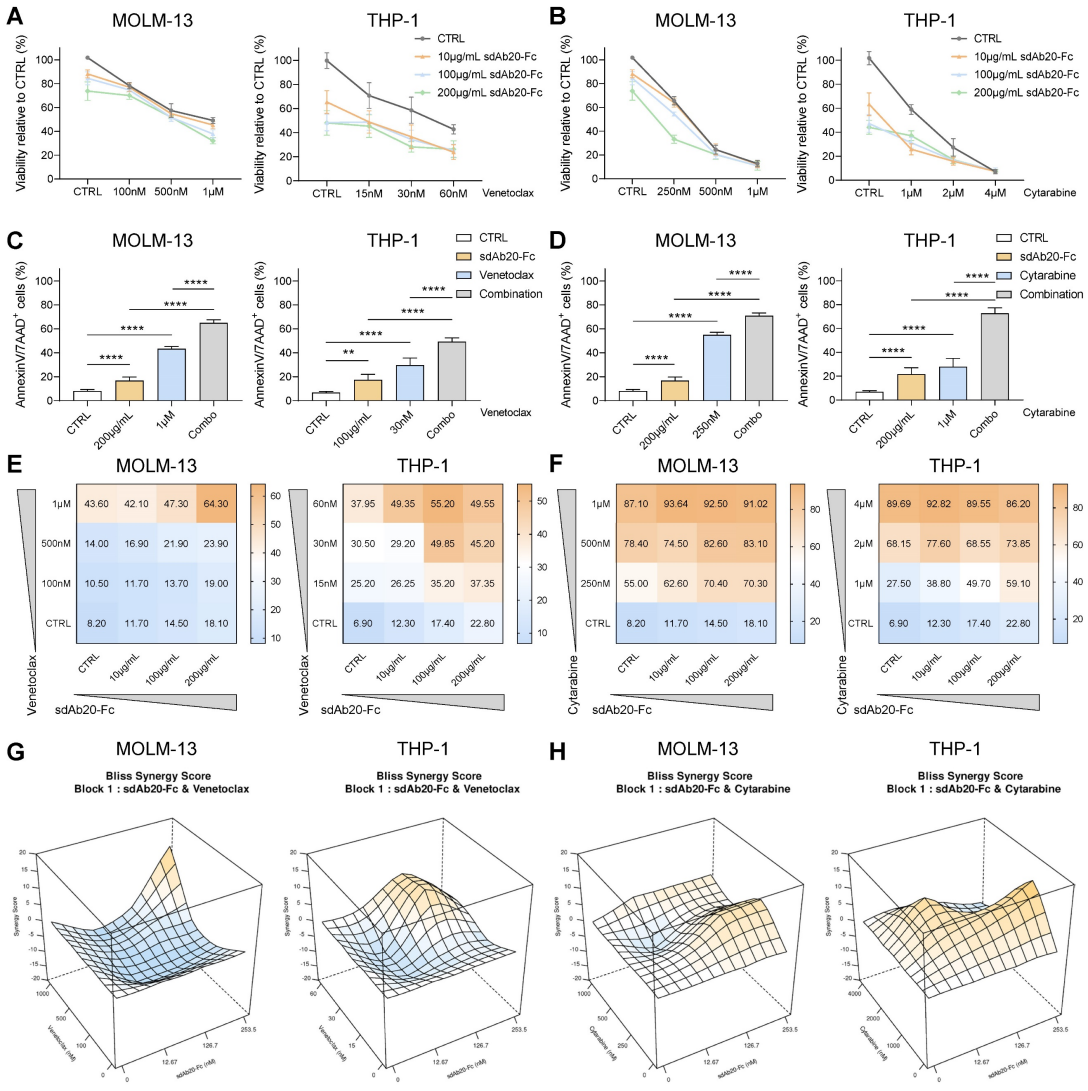
** sdAb20-Fc has synergistic and additive effects when combined with standard-of-care agents cytarabine and venetoclax. (A-D)** THP-1 and MOLM-13 cells were treated with increasing concentrations of sdAb20-Fc in combination with increasing concentrations of cytarabine or venetoclax for 72 h. The effect on cell viability (A-B) and induced apoptosis (C-D) was assessed by CellTiter-Glo viability assay and AnnexinV/7-AAD staining, respectively (MOLM-13: n=5, THP-1: n=6, ± SD). (E-F) Heatmaps display the percentage of apoptotic cells for combination treatment of sdAb20-Fc with venetoclax **(E)** and cytarabine **(F)**. **(G-H)** Synergy of drug interactions was calculated using the BLISS synergy method. Output is generated in 3D format using the SynergyFinder Plus webtool. Data are represented as mean of five samples. p ≤ 0.01 (**) and p ≤ 0.0001 (****) were considered statistically significant. CTRL = control.

Next, we tested the anti-tumor effects of the combination of sdAb20-Fc with cytarabine or venetoclax *in vitro*. The combination of sdAb20-Fc with each agent could clearly reduce cell viability (Figure 7A-B) and significantly increase apoptosis (Figure 7C-D) at indicated concentrations in both THP-1 and MOLM-13 cells. Using the BLISS synergy method, we observed synergistic effects for the sdAb20-Fc/cytarabine combination and additive effects for the sdAb20-Fc/venetoclax combination (Figure 7E-H, Figure S9A-D) in the AML cell lines.

### SdAb20-Fc reduces the cell viability of bone marrow-derived AXL-positive AML samples

**Figure 8:**
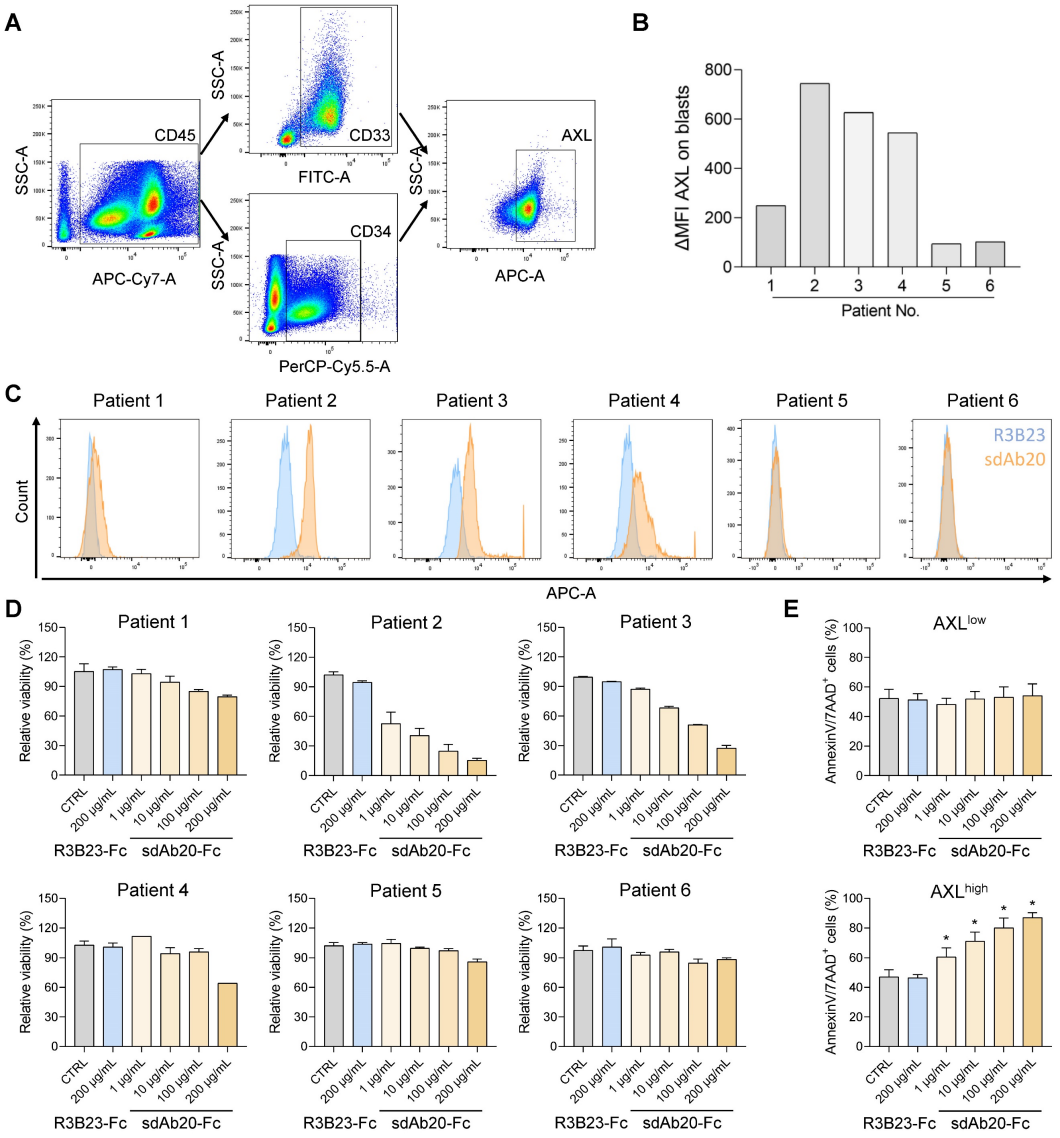
** sdAb20-Fc can specifically alter the viability and apoptosis of primary patient samples. (A)** Gating strategy to determine the number of blasts (based on SSC-A/CD45-A), the percentage of CD33/CD34-positivity and the percentage of AXL-expression in various AML patients.** (B-C)** Flow cytometric assessment of AXL expression in CD33/CD34-positive cells of various primary samples using sdAb20 and the control sdAb R3B23. ΔMFI = MFI (sdAb20) - MFI (CTRL sdAb).** (D-E)** BMMCs of AML patients were treated with R3B23-Fc or increasing concentrations of sdAb20-Fc (10, 100 and 200 µg/mL). The effect on cell viability (D) was assessed using CellTiter-Glo assay (n=2/sample). Apoptosis (E) was measured using AnnexinV/7-AAD staining with flow cytometry. Patients were subdivided into AXL^low^ and AXL^high^, based on their AXL expression (n=3/group, ± SD). p ≤ 0.05 (*) was considered statistically significant. CTRL = control, BMMCs = bone marrow mononuclear cells.

To further validate our findings, we also investigated the effect of sdAb20-Fc on primary bone marrow samples of AML patients at diagnosis or relapse. Using flow cytometry, the percentage of blasts (CD45/SSC), CD33/CD34 positivity and AXL expression (using sdAb20) were determined in six patient samples (Figure 8A-C, Table S3). Based on the median fluorescence intensity (MFI), the patient samples could be subdivided into an AXL^low^ (∆MFI < 300; patients 1, 5 and 6) and an AXL^high^ (∆MFI > 300; patient 2, 3 and 4) group. Mononuclear cells were isolated from the primary bone marrow samples and incubated with sdAb20-Fc or the control R3B23-Fc construct. While sdAb20-Fc reduced the cell viability and increased apoptosis in AXL^high^ samples, this effect could not be observed in the AXL^low^ group (Figure 8D-E). These data again confirm the AXL-specific anti-tumor effects of sdAb20-Fc in AML. Interestingly, the anti-tumor effect of sdAb20-Fc clearly correlated with the AXL-positivity within the blasts (Figure S10A-B).

## Discussion

Although outcomes for young patients with AML have dramatically improved of the past 5 decades, this has not been the case for elderly patients 23. Previous research already identified AXL as a prognostic marker and therapeutic target in AML 6. In this study, we developed AXL-specific sdAbs and evaluated their value for personalized cancer treatment. Biodistribution studies demonstrated the tumor-specific uptake of sdAb20 in THP-1 xenografts and immunocompetent C1498 AML mice. Importantly, the signal in tumor-bearing mice was higher compared to the background signal in naive mice, illustrating its value as a diagnostic tracer in cancer patients. Therapeutically, sdAb20-Fc demonstrated significant anti-tumor effects by inhibiting cell proliferation and cell viability in human AML cell lines and primary patient samples. Moreover, besides its clear single agent anti-tumor effect, our data also demonstrated the therapeutic potential of AXL-specific sdAb20-Fc in combination with the standard-of-care agent cytarabine, which resulted in a synergistic effect.

Receptor tyrosine kinase AXL has been reported to be overexpressed in a variety of human cancers (e.g., lung cancer, breast cancer, colon cancer, melanoma, and pancreatic cancer) and often correlates with a poor prognosis 7, 19. Importantly, the expression of AXL is highly heterogeneous (inter- and intra-patient variability) and is influenced by both the stage of the disease and the administered cancer treatments 6, 11. Previous work by Wang and colleagues already demonstrated the potential of ^64^Cu-labeled anti-human AXL antibodies to visualize AXL^+^ tumors and monitor the effect of anti-AXL therapies in triple negative breast cancer (TNBC)-bearing nude mice 24. However, since AXL is expressed in multiple immune cells (e.g., macrophages, natural killer cells) 20, the evaluation in immunocompetent mice and the use of murine AXL-targeting Abs is indispensable to realistically gauge the background signal in the tissue of interest. Moreover, because of their small size, rapid biodistribution, homogeneous tumor labeling, rapid renal clearance and low dosimetry, we preferred the development of sdAbs to generate high-contrast images *in vivo* early after administration 25-27. We found that lead compound sdAb20 could better distinguish AXL^+^ from AXL^-^ AML tumors compared to mAbs using flow cytometry. Moreover, the signal of ^99m^Tc-labeled sdAb20 was clearly higher in tumor-bearing C1498 mice compared to naive mice, which was also visible on the generated SPECT/CT images. Co-injection of unlabeled sdAb20-Fc with ^99m^Tc-labeled sdAb20 could significantly improve the tumor uptake and visualization in THP-1 xenografts by reducing the splenic background. This was further explained by assessing the biodistribution profile of ^111^In-labeled sdAb20-Fc, which demonstrated increased uptake in the spleen and low tumor accumulation after 24 h. A similar co-injection approach was previously reported as a useful method for PET imaging of ^64^Cu-labeled anti-PD-L1 antibodies in orthotopic pancreatic cancer models 28. In the study of Movahedi *et al.,* an excess of unlabeled, bivalent anti-MMR sdAbs were co-injected with monovalent anti-MMR sdAbs to reduce sdAb accumulation in extra-tumoral organs to background levels, without compromising tumor uptake 29.

It is important to emphasize that AXL is not only expressed by malignant cancer cells, but can also be located on the surface of various other cells, including immune cells. Therefore, we specifically developed cross-reactive sdAbs, targeting both murine and human AXL protein, allowing us to demonstrate that even in the presence of an immunocompetent environment and AXL^+^ non-AML cells, we were still able to distinguish the specific tumor uptake from the background levels using SPECT/CT imaging, fostering the clinical potential of sdAb20.

It is also important to mention that the biodistribution experiment illustrated a high kidney uptake of ^99m^Tc-labeled sdAbs. This could be explained by their small size together with receptor-mediated endocytosis of these charged molecules in the kidney proximal tubuli cells with the number of polar residues in the C-terminal amino acid tag, which are the predominant reasons for sdAb-associated kidney retention 30. The use of untagged sdAbs or the co-injection with plasma expander gelofusin are two known methods to significantly reduce the kidney uptake of radiolabeled sdAbs 30-32. In comparison, the increased size of sdAb20-Fc (~ 80 kDa) resulted in a lower kidney uptake (6.48 ± 2.46 %IA/g) compared to sdAb20 (210.84 ± 64.25 %IA/g), related to the slower renal clearance. Furthermore, the use of ^99m^Tc as a diagnostic tracer is particularly of interest for imaging in animal models (e.g. easy His6-tag based coupling, SPECT/CT imaging), but should be replaced and tested with a radionuclide that emits positrons, like Gallium-68, Fluor-18 or Zirconium-89, for PET imaging 33. In patients, PET imaging is preferred based on its improved image quality and spatial resolution, higher diagnostic accuracy and lower patient dosimetry 34.

AXL's pleiotropic role in cell survival, drug resistance and immune suppression makes it a promising target for cancer therapy 8. The past years, diverse AXL-targeting agents have been developed, including small molecule inhibitors (e.g., bemcentinib, bosutinib, gilteritinib, cabozantinib), anti-AXL mAbs (e.g., tilvestamab, YW327.6S2), antibody-drug conjugates (e.g., AXL-107-MMAE), nucleotide aptamers (e.g., GL21.T) and soluble receptors (e.g., AXL decoy receptor) 7, 8. Although these AXL-targeted therapies appear promising, they often induce off-target effects through the inhibition of additional kinases. This may lead to subsequent unexpected toxicities and limit their clinical use 8. In our study, the AXL inhibitor bemcentinib, that was included as a positive control, clearly demonstrated also AXL-independent anti-tumor effects *in vitro*. This aspect has previously been reported by Zdzalik-Bielecka *et al.* and was linked to bemcentinib-mediated targeting of the endo-lysosomal system and autophagy pathway, in an AXL-independent manner, in glioblastoma cells 35. In a therapeutic context, sdAbs also possess desirable properties compared to mAbs, such as their low immunogenicity risk 13, modularity, and compact size, making them suitable for targeting recessed epitopes. Coupling of sdAb20 to an Fc tail increased its *in vitro* GAS6/AXL blocking capacity, while maintaining half the molecular weight of a conventional antibody. SdAb20-Fc treatment of AML cell lines and patient samples resulted in multiple anti-tumor effects *in vitro*, including reduced cell proliferation and viability, and increased apoptosis. Since GAS6/AXL interaction has been described to trigger various signaling pathways in cancer cells, including PI3K-AKT, NFκB, RAS-MEK-ERK, SRC-FAK and JAK-STAT 8; it might be interesting to further elucidate the downstream mechanisms of action of sdAb20-Fc in AML.

Current clinical trials focus on the combination of AXL inhibitors with chemotherapeutic agents and immunotherapies in cancer 8, 36-38. AXL inhibitors have been described to increase sensitivity towards standard-of-care agents venetoclax or cytarabine in AML 39, 40. However, a direct comparison to identify the most promising combination therapy is missing. In concordance with previous studies, we observed increased anti-tumor effects combining sdAb20-Fc with either venetoclax or cytarabine. Interestingly, we found that cytarabine could induce AXL expression, and that the combination with sdAb20-Fc resulted in synergistic anti-AML effects. On the other hand, venetoclax had no effect on AXL expression and the combination with sdAb20-Fc resulted in additive effects in AML cell lines.

Altogether, this study describes the development and theragnostic potential of AXL-specific sdAbs in AML. Radiolabeled sdAb20-based imaging may be an accurate and non-invasive method to monitor AXL expression in cancer patients, paving the way for personalized treatment and increased effectiveness of AXL inhibitors. Our study also fosters the evaluation of sdAb20-Fc in combination with cytarabine in preclinical models for AML. Future PK/PD and toxicological studies are still required to determine the dose and safety profile of sdAb20 and sdAb20-Fc for first-in-human clinical trials.

## Supplementary Material

Supplementary materials and methods, figures and tables.

## Abbreviations

%IA/g: percentage injected activity per gram organ
^111^In: indium-111
2-ME: 2-mercapto-ethanol
7-AAD: 7-aminoactinomycin D
^99m^Tc: technetium-99m
AF647: alexa fluor 647
AKT: protein kinase B
AMIDE: a medical image data examiner
AML: acute myeloid leukemia
ANOVA: analysis of variance
APC: allophycocyanin
ATCC: american type culture collection
AXL: anexelecto
BCA: bicinchronic acid
BCL-2: B-cell lyphoma 2
BM: bone marrow
BrdU: bromodeoxyuridine
BSA: bovine serum albumin
BV605: briljant violet 605
CCA: cell cycle arrest
CD33: cluster of differentiation 33
CDR: complementarity-determining region
CRC: colorectal cancer
CT: computed tomography
DMEM: dulbecco's modified eagle medium
DMSO: dimethylsulfoxide
ECL: enhanced chemiluminescent substrate
EDC: 1-ethyl-3-(3-dimethylaminopropyl)carbodiimide
EDTA: ethylenediaminetetraacetic acid
ELISA: enzyme-linked immunosorbent assay
ERK: extracellular signal-regulated kinases
FACS: fluorescence-activated cell sorting
FAK: protein tyrosine kinase 2
FCS: forward scatter
FDA: food & drug administration
FITC: fluorescein isothiocyanate
GAS6: growth arrest specific factor 6
HA: hemagglutinin
HBS: hepes-buffered saline
IMAC: immobilized metal affinity chromatography
IMDM: iscove's modified dulbecco's medium
IMGT: ImMunoGeneTics
iTLC: instant thin-layer chromatography
JAK: janus kinase
kV: kilo volt
mAb: monoclonal antibody
MBq: mega becquerel
MEK: mitogen-activated protein kinase kinase
MFI: median fluorescence intensity
mIgG2a: mouse immunoglobuline G2a
NF-kB: nuclear factor-kappa B
NHS: n-hydroxy-succinimide
NSCLC: non-small cell lung carcinoma
PBS: phosphate-buffered saline
PCR: polymerase chain reaction
PD-L1: programmed death ligand 1
PE: phycoerythrin
PET: positron emission tomography
PI3K: phosphoinositide 3-kinase
PK/PD: pharmacokinetic/pharmacodynamic
qRT-PCR: quantitative reverse transcription polymerase chain reaction
RAS: rat sarcoma virus
RPMI: roswell park memorial institute
SCID: severe combined immunodeficiency
SD: standard deviation
sdAbs: single domain antibodies
SDS-PAGE: sodium dodecyl sulfate polyacrylamide gel electrophoresis
SEC: size exclusion chromatography
SPECT: single-photon emission computed tomography
SRC: proto-oncogene tyrosine-protein kinase Src (Sarcoma)
SSC: side scatter
STAT: signal transducer and activator of transcription
TNBC: triple-negative breast cancer
Tm: melting temperature
