# Supplementary material for: AXL-specific single domain antibodies show diagnostic potential and anti-tumor activity in Acute Myeloid Leukemia
Source: Theranostics. 2024 Apr 15;14(7):2656–74. doi: 10.7150/thno.91456 (PMC11103505; doi:10.7150/thno.91456)
Supplement: Supplementary file 1 — Supplementary materials and methods, figures and tables. [file thnov14p2656s1.pdf]

SUPPLEMENTARY MATERIALS AND METHODS

Cell Culture

The human AML cell lines KG-1a, OCI-AML3, MOLM-13, MV-4-11 and THP-1, the murine AML cell line C1498, the human colorectal cancer (CRC) cell line HCT-116 and the human non-small cell lung carcinoma (NSCLC) cell line A549 were obtained from ATCC (Molsheim, France). KG-1a, OCI-AML3, MOLM-13 and HCT-116 cells were cultured in Roswell Park Memorial Institute 1640 medium (RPMI-1640; Gibco; Thermo Fisher Scientific), 10% fetal calf serum (FCS) (PAN Biotech, Aidenbach, Bayern, Germany), 100 U/mL penicillin, 100 µg/mL streptomycin, and 2 mM L-glutamine (Lonza, Basel, Switzerland). For the THP-1 cell line, 2-mercapto-ethanol (2-ME) was also added to a final concentration of 0.05 mM. MV-4-11 and C1498 cells were cultured in Iscove's Modified Dulbecco's Medium (IMDM; Gibco; Thermo Fisher Scientific), supplemented with 10% FCS, 100 U/mL penicillin, 100 µg/mL streptomycin and 2mM L-glutamine. For the MV-4-11 cell line, 100 U/mL penicillin and 100 µg/mL streptomycin were added to the culture medium. A549 cells were cultured in Dulbecco's Modified Eagle's Medium (DMEM; Gibco; Thermo Fisher Scientific), with 10% FCS, 100 U/mL penicillin, 100 µg/mL streptomycin and 2 mM L-glutamine. All cell lines were cultured at 37°C in 5% CO_2, were regularly tested for mycoplasma contamination using the MycoAlert Mycoplasma Detection Kit (Lonza, USA, #LT07-418) and were passaged no more than one month prior to experiments.

Lentiviral transduction of cells

The murine *Axl* gene was cloned into a transfer plasmid (pHR') using Gibson cloning. In short, gBlocks were designed to encode the sequence for mus musculus AXL receptor tyrosine kinase (*Axl*), transcript variant 1 (Gene ID: 26362; NM_009465.4) and purchased from Integrated DNA Technologies (Leuven, Belgium). Lentiviral vectors were produced by transfection of HEK293T cells with the transfer plasmid, an envelope encoding plasmid (pMD.G) and a packaging plasmid (pCMVΔR8.9), as previously described [1]. The pMD.G and pCMVΔR8.9 plasmids were both provided by D. Trono, Geneva, Switzerland. Lentiviral particles were harvested 48 and 72 h following transfection and concentrated using

ultracentrifugation. Subsequently, murine C1498 AML cells were transduced with lentiviral vectors harbouring mouse *Axl* at a ratio of 10 transducing lentiviral particles per cell (multiplicity of infection 10). Transduced cells were then subjected to magnetic-activated cell sorting, using an APC-conjugated mouse AXL antibody (R&D Systems, FAB8541A) and anti-APC microbeads (Miltenyi Biotec, 130-090-855) to increase the purity of AXL-expressing C1498 cells, further referred to as AXL^{high}. Non-transduced cells are referred to as AXL^{low}.

Thermal stability assay

The melting temperature (Tm) values of anti-AXL sdAbs and their controls were determined using a ThermoFluor® assay, using CFX Connect™ Real-Time PCR (Biorad, Pleasanton, CA, USA) and the fluoroprobe Sypro® Orange dye (Molecular Probes, Oregon, OR, USA), as previously described [2]. For thermal denaturation, the samples were heated from 10 to 95 °C, with a gradual increase of 0.5 °C per 30 s, and a 10 s hold step at every checkpoint, followed by fluorescence reading. The Tm values were subsequently calculated using the Boltzmann equation.

Competition assay

The blocking capacity of anti-AXL sdAb20 and sdAb20-Fc was determined using the Biacore-T200 device (GE Healthcare). The measurements were performed at 25°C using HBS as running buffer. First, GAS6 (R&D Systems, #885-GSB) was immobilized on a CM5 sensor chip using linkage chemistry with EDC and NHS, and remaining free EDC-NHS linkers were neutralized with 1 M ethanolamine-HCl pH 8.5. Afterwards, a serial dilution of sdAb20, sdAb20-Fc and the control sdAbs R3B23 and R3B23-Fc (10,000 to 0.9766 nM) was made and incubated with 150 nM recombinant AXL protein in a 1:1 ratio for 1 h at room temperature. Recombinant AXL protein without sdAbs was used as a positive control. The dilutions were analyzed at a flow rate of 10 µL/min and after each cycle, the chip was regenerated for 30 s using 100 mM Glycine HCl pH 3.0. The IC_{50} value of the sdAbs was calculated by comparing the

response units of the dilution to the blank (HBS only) and the positive control (AXL protein only). Based on these results, a nonlinear regression dose-response curve was generated using GraphPad Prism.

Protein Structure Prediction

Protein structural predictions were generated using AlphaFold2 [3], accessed using AlphaFold Colab. Coupled with Google Colaboratory, the open-source software ColabFold [4] allows accelerated prediction of protein structures and protein-protein interactions by combining the fast homology search of MMseqs2 (UniRef + Environmental) with AlphaFold2. The sequences used for structural predictions can be found in Table S1 and were retrieved from the UniProt database (#P30530, #Q00993, #Q14393 and #Q61592). Structural visualization and alignments were generated using the PyMOL Molecular Graphics System, Version 2.5.2, license invoice #51791 (Schrödinger, Mannheim, Germany).

Flow cytometry

To evaluate the cell binding capacity of sdAb20, 200 nM sdAb was pre-incubated with 1 µL mouse anti-histidine tag antibody (Bio-Rad Laboratories, #MCA1396) in PBS supplemented with 0.5% bovine serum albumin and 0.05% sodium-azide for 1 h on ice at 4°C. In the meantime, cells were counted and washed with cold PBS, after which the cells were incubated with the sdAb20-anti-histidine tag mix for 1 h on ice at 4°C. Next, cells were washed with cold PBS to remove unbound sdAb20 and incubated with 1 µL APC-conjugated rat anti-mouse IgG1 antibody (Biolegend) for 30 min on ice.

To investigate the cell binding potential of sdAb20-Fc, we directly fluorescently labeled sdAb20-Fc using the Alexa Fluor^{TM} 647 Protein Labeling Kit (Invitrogen Carlsbad, CA). Cells were first washed and incubated for 30 min with FcR blocking reagent (Miltenyi Biotec, Leiden) (Fc-block) at 4°C on ice. Afterwards, the cells were stained with the labeled sdAb20-Fc construct for 1 h at 4°C on ice.

An APC-conjugated mouse AXL antibody (FAB8541A) and human AXL antibody (FAB154A) (R&D Systems) were used as positive controls. The anti-human monoclonal antibodies CD45-APC-Cy7 (#368516), CD33-FITC (#366620) and CD34-PerCP-Cy5.5 (#343522) that were used for the identification

and quantification of AML blasts in primary AML patient samples were purchased from Biolegend and used according to the manufacturer's protocol. All antibodies were compared to their isotype control and sdAb20 was compared to the irrelevant sdAb R3B23. Flow cytometry was performed on the FACSCanto, LSRFortessa and FACSCelesta flow cytometers (BD Biosciences, Belgium). Data were processed using BD FACSDiva™ (BD Biosciences) and FlowJo 10.9® software (Tree Star; Inc., Ashland, OR, USA).

RNA isolation and quantitative Real-Time PCR

RNA was extracted and purified using the NucleoSpin RNA Plus Kit (Macherey-Nagel, Düren, Germany), according to the manufacturer's protocol and RNA concentration was measured with Nanodrop™ (Thermo Fisher Scientific). Next, 500 ng/µL RNA was converted into cDNA using the Verso cDNA Synthesis Kit (Thermo Fisher Scientific). Real-time PCR was performed in a volume of 25 µL with 1 µL primer mix, 10.5 µL nuclease-free water, 1 µL cDNA, and 12.5 µL SYBR Green (Applied Biosystems, Thermo Fisher Scientific) using the Quantstudio 12 K Flex Real-Time PCR System (Thermo Fisher Scientific). Gene-specific primers were purchased from Integrated DNA Technologies (Leuven, Belgium) and were used at a final concentration of 200 nM. Primer sequences are listed in Table S2. Primers for human GAPDH were obtained from Qiagen (#QT00079247) (Westburg, Leusden, Netherlands) and was used as a housekeeping gene for data normalization. Differential gene expression levels were determined using the comparative ΔΔCt method.

Western blot analysis

Cells were seeded at 500,000 cells/mL, collected after 24 h and immediately lysed for 10 min using lysis buffer containing 50 mM Tris (pH 7.6), 150 mM NaCl, 1% Nonidet P40, and 0.25% sodium deoxycholate. The following protease and phosphatase inhibitors were added: 4 mM Na_3VO_4, 1 mm $Na_4P_2O_7$, 2 µg/mL aprotinin, 50 µg/mL leupeptin, 500 µg/mL trypsin inhibitor, 10 µM benzamidine, 2.5 mM para-nitrophenyl benzoate (all from Sigma-Aldrich); 50 mM NaF, 5 mM ethylenediaminetetraacetic acid

(both from VWR International, Radnor, PA, USA); and 1 mM 4-(2-aminoethyl) benzene sulfonyl fluoride hydrochloride and 50 µg/mL pepstatin A (both from ICN Biomedicals, Costa Mesa, CA, USA). After centrifugation, protein concentrations were determined in the supernatant using the Pierce™ Bicinchoninic Acid (BCA) Protein Assay Kit (Thermo Fisher Scientific). An equal volume of 2x Laemmli Sample Buffer (Bio-Rad) was added to 30 µg of protein and the samples were heated for 5 min at 95°C. Afterward, the samples were separated using a 10-12.5% sodium dodecyl sulfate-polyacrylamide gel and transferred onto a polyvinylidene difluoride membrane (Bio-Rad). The blots were blocked using 5% low-fat milk in TBS with 0.1% Tween® 20 detergent (TBST) buffer (50 mM Tris-HCl; 150 mM NaCl; pH 7.4) for 1 h at room temperature, followed by overnight incubation with primary antibodies at 4°C: P-AXL (#5724S, 1/500), AXL (#8661, 1/1000), GAS6 (#67202S; 1/1000). β-Actin (#4967L, 1:1000) was used as a loading control. After washing, the blots were incubated with Horseradish peroxidase (HRP)-conjugated secondary antibodies (diluted 1:1000) for 1 h at room temperature. All antibodies were purchased from Cell Signaling Technology (Leiden, The Netherlands). Data were collected using enhanced chemiluminescence (ECL) and an Odyssey Fc Imager (LI-COR Biosciences, Bad Homburg, Germany).

1. Breckpot K, Aerts JL, Thielemans K. Lentiviral vectors for cancer immunotherapy: transforming infectious particles into therapeutics. Gene Ther. 2007; 14: 847-62.
2. Boivin S, Kozak S, Meijers R. Optimization of protein purification and characterization using Thermofluor screens. Protein Expr Purif. 2013; 91: 192-206.
3. Jumper J, Evans R, Pritzel A, Green T, Figurnov M, Ronneberger O, et al. Highly accurate protein structure prediction with AlphaFold. Nature. 2021; 596: 583-9.
4. Mirdita M, Schutze K, Moriwaki Y, Heo L, Ovchinnikov S, Steinegger M. ColabFold: making protein folding accessible to all. Nat Methods. 2022; 19: 679-82.

UniProt ID code	Protein	Sequence
**P30530**	AXL – Homo sapiens	MAPRGTQAEESPFVGNPGNITGARGLTGTLRCQLQVQGEPPEVHWLRDGQILELADSTQTQVPLGEDEQDDWIVVSQLRITSLQLSDTGQYQCLVFLGHQTFVSQPGYVGLEGLPYFLEEPEDRTVAANTPFNLSCQAQGPPEPVDLLWLQDAVPLATAPGHGPQRSLHVPGLNKTSSFSCEAHNAKGVTTSRTATITVLPQQPRNLHLVSRQPTELEVAWTPGLSGIYPLTHCTLQAVLSDDGMGIQAGEPDPPEEPLTSQASVPPHQLRLGSLHPHTPYHIRVACTSSQGPSSWTHWLPVETPEGVPLGPPENISATRNGSQAFVHWQEPRAPLQGTLLGYRLAYQGQDTPEVLMDIGLRQEVTLELQGDGSVSNLTVCVAAYTAAGDGPWSLPVPLEAWRP
**BV506**	Axl – Mus musculus	MGRVPLAWWLALCCWGCAAHKDTQTEAGSPFVGNPGNITGARGLTGTLRCELQVQGEPPEVVWLRDGQILELADNTQTQVPLGEDWQDEWKVVSQLRISALQLSDAGEYQCMVHLEGRTFVSQPGFVGLEGLPYFLEEPEDKAVPANTPFNLSCQAQGPPEPVTLLWLQDAVPLAPVTGHSSQHSLQTPGLNKTSSFSCEAHNAKGVTTSRTATITVLPQRPHHLHVVSRQPTELEVAWTPGLSGIYPLTHCNLQAVLSDDGVGIWLGKSDPPEDPLTLQVSVPPHQLRLEKLLPHTPYHIRISCSSSQGPSPWTHWLPVETTEGVPLGPPENVSAMRNGSQVLVRWQEPRVPLQGTLLGYRLAYRGQDTPEVLMDIGLTREVTLELRGDRPVANLTVSVTAYTSAGDGPWSLPVPLEPWRPGQGQPLHHLVSEPPPRAFSWPWW
**Q14393**	GAS6 – Homo sapiens	GRMFSGTPVIRLRFKRLQPTRLVAEFDFRTFDPEGILLFAGGHQDSTWIVLALRAGRLELQLRYNGVGRVTSSGPVINHGMWQTISVEELARNLVIKVNRDAVMKIAVAGDLFQPERGLYHLNLTVGGIPFHEKDLVQPINPRLDGCMRSWNWLNGEDTTIQETVKVNTRMQCFSVTERGSFYPGSGFAFYSLDYMRTPLDVGTESTWEVEVVAHIRPAADTGVLFALWAPDLRAVPLSVALVDYHSTKKLKKQLVVLAVEHTALALMEIKVCDGQEHVVTVSLRDGEATLEVDGTRGQSEVSAAQLQERLAVLERHLRSPVLTFAGGLPDVPVTSAPVTAFYRGCMTLEVNRRLLDLDEAAYKHSDITAHSCPPVEPAAA
**Q61592**	Gas6 – Mus musculus	GRMFSGTPVIRLRFKRLQPTRLLAEFDFRTFDPEGVLFFAGGRSDSTWIVLGLRAGRLELQLRYNGVGRITSSGPTINHGMWQTISVEELERNLVIKVNKDAVMKIAVAGELFQLERGLYHLNLTVGGIPFKESELVQPINPRLDGCMRSWNWLNGEDSAIQETVKANTKMQCFSVTERGSFFPGNGFATYRLNYTRTSLDVGTETTWEVKVVARIRPATDTGVLLALVGDDDVVPISVALVDYHSTKKLKKQLVVLAVEDVALALMEIKVCDSQEHTVTVSLREGEATLEVDGTKGQSEVSTAQLQERLDTLKTHLQGSVHTYVGGLPEVSVISAPVTAFYRGCMTLEVNGKILDLDTASYKHSDITSHSCPPVEHATP

Supplementary Table 1. Sequences used for protein structure prediction using AlphaFold2.

Gene		Primer sequence 5'-3'
Human *GAPDH*	Forward	GTC TCC TCT GAC TTC AAC AGC G
	Reverse	ACC ACC CTG TTG CTG TAG CCA A
Mouse *Gapdh*	Forward	GTG GCA AAG TGG AGA TTG TTG
	Reverse	CGT TGA ATT TGC CGT GAG TG
Human *AXL*	Forward	AGC CCT GTC TTC CTA CCT ATC
	Reverse	AGA TGG TGA TGC TAC TGC TTT C
Mouse *Axl*	Forward	CAA GGG ATC GTC TCA CAT CTT
	Reverse	CAT CTC GGT CCA AAG ACT CAT C
Human *GAS6*	Forward	TCT GTG GCA CTG GTA GAC TAT
	Reverse	CGC AGA CCT TGA TCT CCA TTA G
Mouse *Gas6*	Forward	CGG CAT GTG GCA AAC TAT CT
	Reverse	CAG CTA CCG CGA TCT TCA TTA C

Supplementary Table 2. Primer sequences used for realtime PCR analysis.

Patient	Age	Gender	Diagnosis	Origin	Blasts (% flow)	Blasts (% morfo)	CD33/CD34
1	54	M	NPM1mut/AML-M5	BM	90%	91%	CD34-/CD33++
2	71	F	AML-M4	BM	21%	41%	CD34-/CD33++
3	76	M	AML-M0	BM	95%	92%	CD34+/CD33+
4	72	M	AML-M2	BM	40%	54%	CD34+/CD33+
5	76	M	AML-M1	BM	44%	37%	CD34+/CD33weak+
6	66	F	AML-inv (16), KITmut	BM	49.5%	64%	CD34+/CD33weak+

Supplementary Table 3. AML patient's characteristics.

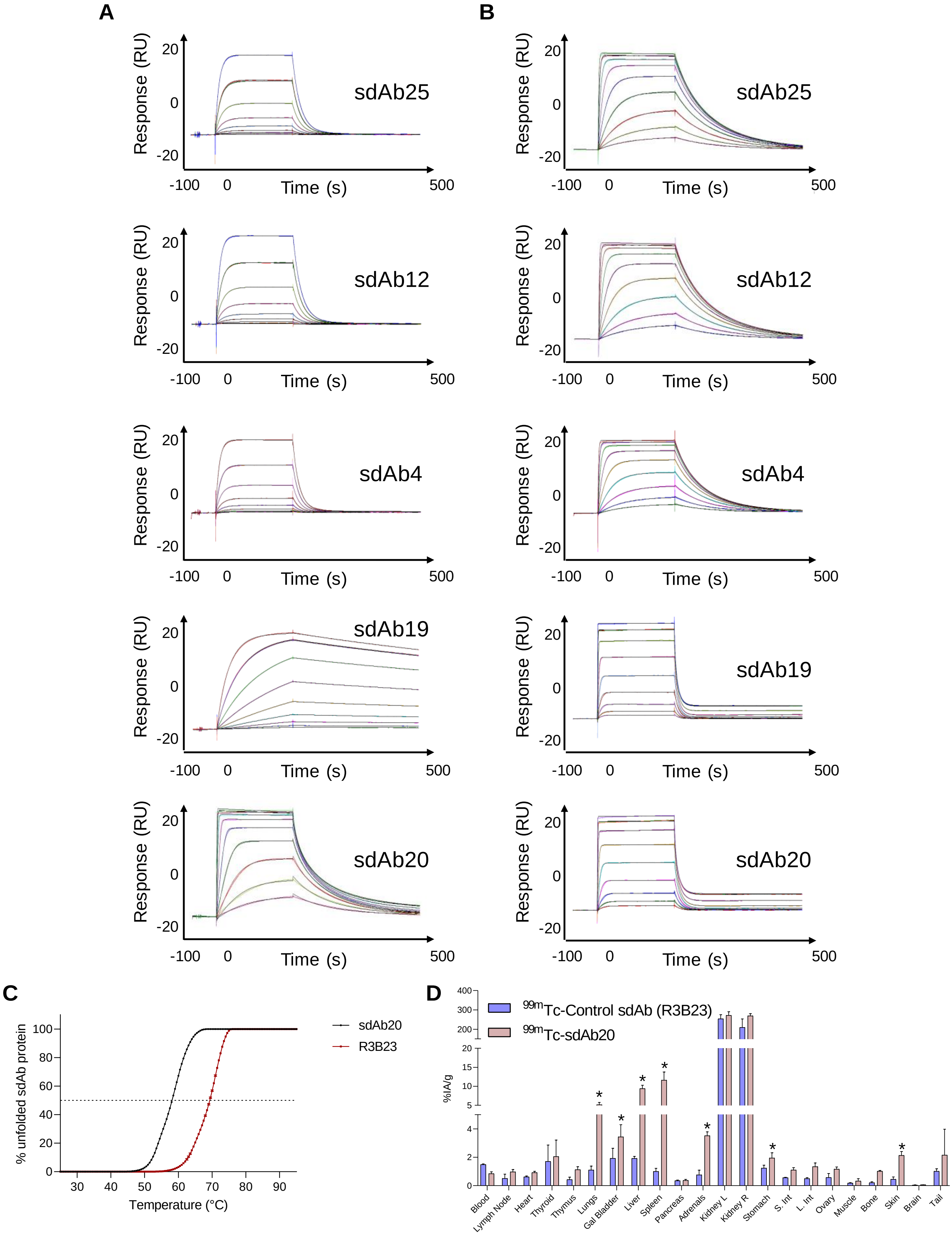

Supplementary Figure 1. (A-B) Shown are Biacore-T200 sensorgrams obtained for the binding of anti-AXL sdAbs to immobilized recombinant mouse (A) and human (B) AXL protein, after instantaneous background depletion. The sdAbs were injected at ten different concentrations (shown in different colors), ranging from 1.9 to 500 nM. **(C)** Percentage unfolded sdAb20 and R3B23 at different temperatures, determined by the ThermoFluor® assay. **(D)** Graphical representation of the ex vivo biodistribution profile of R3B23 and sdAb20 in naive CB17-SCID mice. Organs were isolated 90 minutes post-injection of radiolabeled (^{99m}Tc) sdAb20 or R3B23 control tracer (n=3). Results are presented as mean %IA/g ± SD. $p \leq 0.05$ (*) was considered statistically significant.

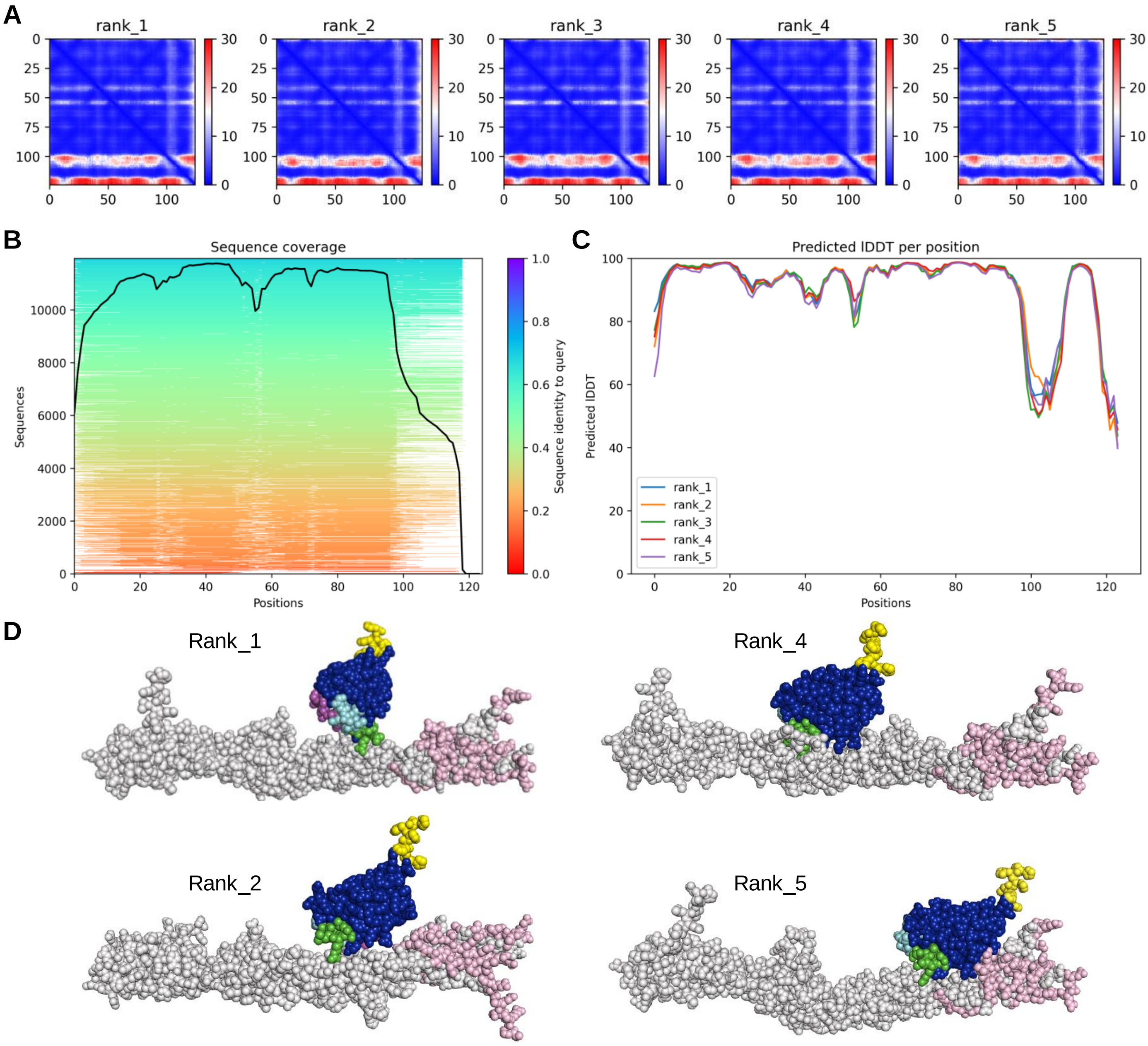

Supplementary Figure 2. (A) Prediction aligned error (PAE) score for models ranked 1 to 5. This score quantifies the discrepancy between the predicted distances for pairs of amino acid residues. The x and y axes of the graph represent the positions of individual amino acids. The level of uncertainty in the predicted distance between two amino acids is depicted by a color gradient ranging from blue (0 Å) to red (30 Å), as indicated in the accompanying legend. The color at the intersection of a vertical line originating from one amino acid's position on the x-axis and a horizontal line from another amino acid's position on the y-axis represents the error in the predicted distance between these two residues. PAE graphs consistently exhibit a diagonal blue line, reflecting the fact that amino acids closely positioned in the primary sequence are also proximate in the 3D structural arrangement. **(B)** Number of homologous sequences identified per position – 30 to 100 sequences are required for best performance prediction. **(C)** Predicted local distance difference test (LDDT) score per position for the five models generated by Alphafold2. The graph illustrates the relationship between amino acid positions and their corresponding predicted LDDT scores. When pLDDT values exceed 90, it suggests a very high level of accuracy, equivalent to experimentally determined structures. Conversely, values falling within the range of 50 to 70 indicate lower accuracy, but it is probable that the predictions for individual secondary structures remain correct. **(D)** Alternative AlphaFold2 predicitons of the interaction between sdAb20 and AXL. Rank_3 is shown in Figure 1.

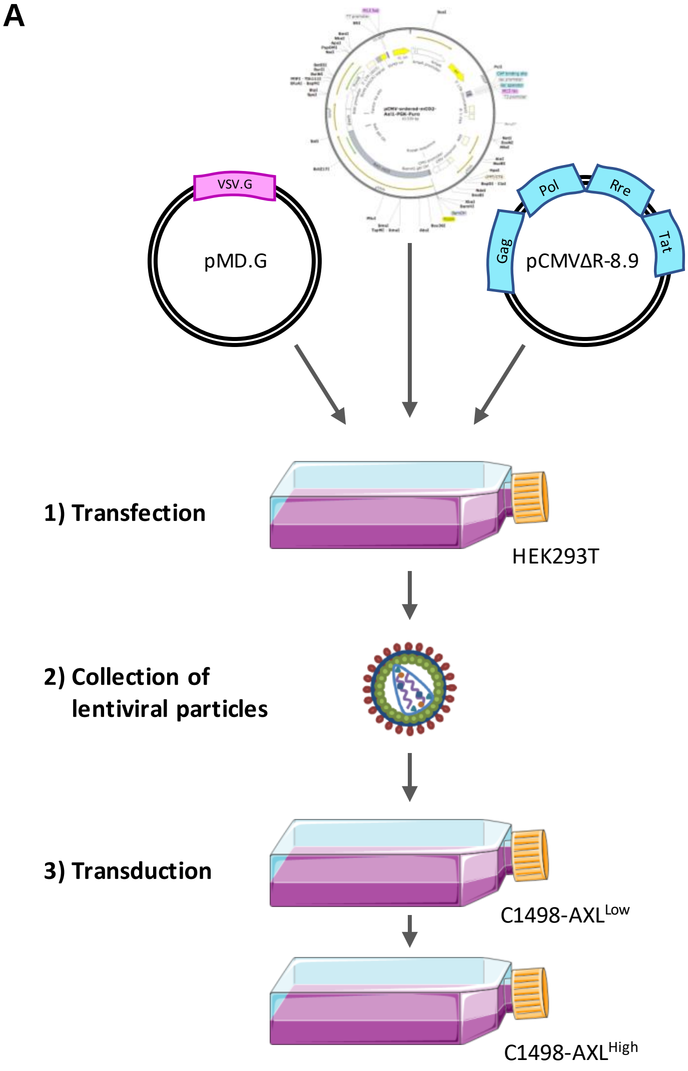

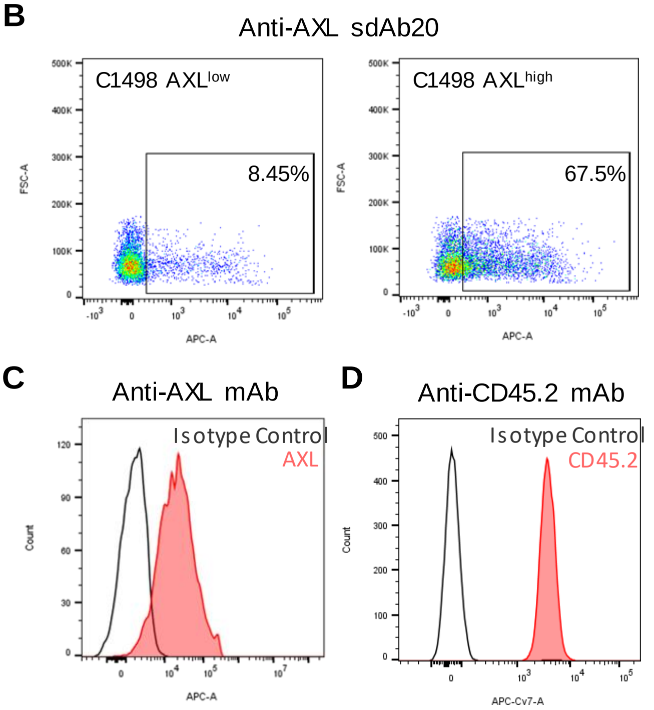

Supplementary Figure 3. (A) Schematic representation of the lentiviral transduction of C1498 cells. HEK293T cells were lentivirally transfected with the third generation pCMV-ordered-mCO2-Axl1-PGK-PURO vector, expressing the first variant of the AXL1 coding sequence, together with an envelope-expressing plasmid (pMD.G) and a packaging plasmid (pCMV delta R8.9). Subsequently, harvested lentiviral particles were used to transduce C1498-AXL^{low} cells. **(B-C)** C1498 cell transduction was confirmed by flow cytometry using sdAb20-APC (B) and a conventional APC-labeled mAb (C) (n=5). **(D)** Flow cytometric confirmation of the expression of end-stage tumorload marker CD45.2 on AXL-positive C1498 cells (n=3).

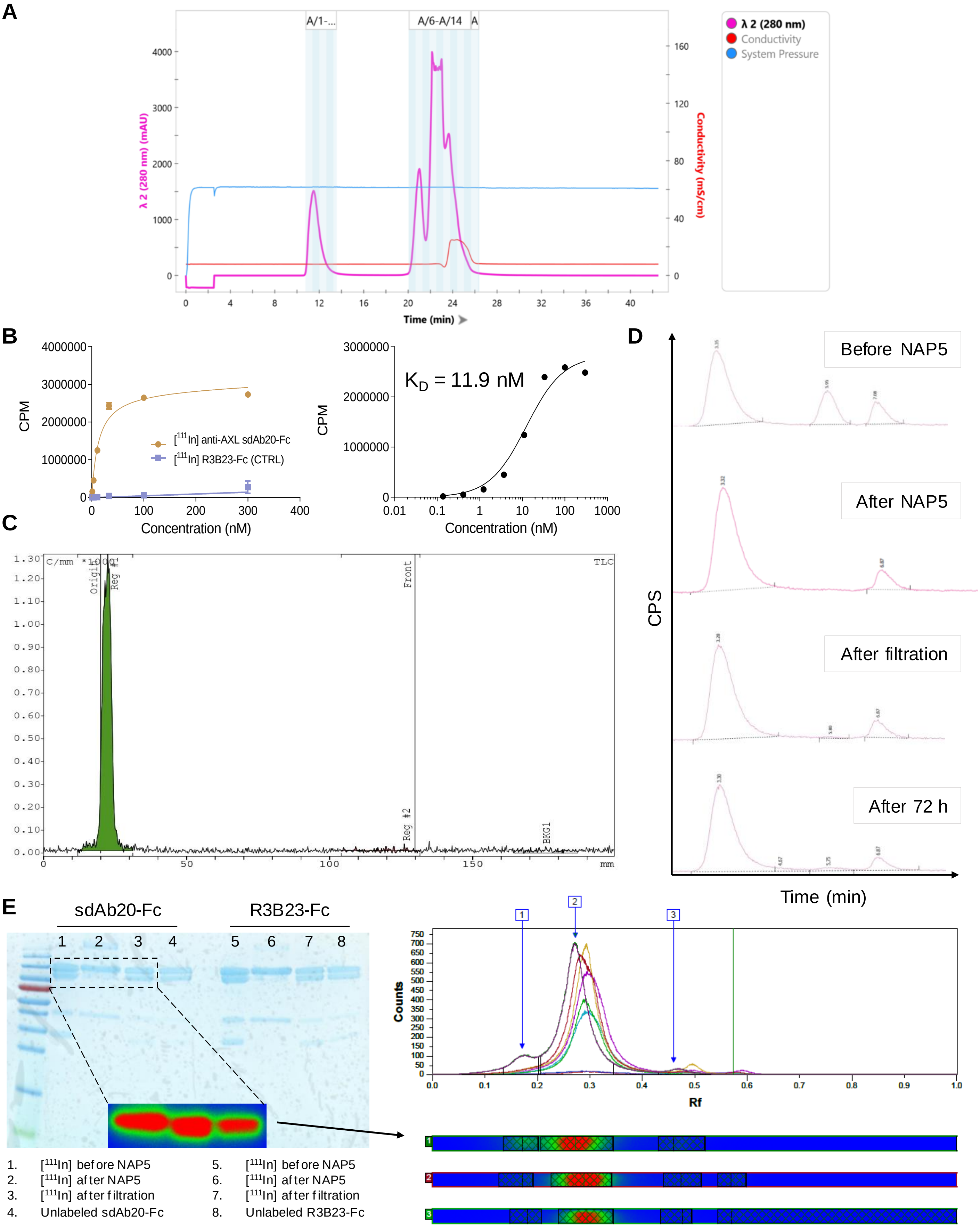

Supplementary Figure 4. (A) Size exclusion chromatography (SEC) confirms the addition of at least one DTPA molecule to sdAb20-Fc as indicated by the near-complete shift of the peak from 12 to 22 min, as measured at a wavelength of 280 nm. **(B)** Saturation binding curve of [^{111}In]In-DTPA-sdAb20-Fc on recombinant mouse Axl antigen using radio-ELISA, confirming the intact functionality of the construct. Specific binding (KD) was calculated as the difference between total and non-specific binding. Data are expressed as mean ± SD. **(C)** Instant thin layer chromatography (iTLC) of [^{111}In]In-DTPA-sdAb20-Fc demonstrated high radiochemical purity. Radioactivity (green) chromatograms revealed greater than 90% of the radioactivity at the origin (Reg #1). Unbound, free ^{111}In, is visualized as the small red bump at region 2 (Reg #2) and moves with the solvent front. **(D)** SEC chromatograms confirmed the successful radiolabeling of ^{111}In to the DTPA-sdAb20-Fc construct, especially after NAP-5 column purification. The construct remained stable up to at least 72 h. All SEC profiles are aligned at the main [^{111}In]In-DTPA-sdAb20-Fc peak. **(E)** The purity of the sdAbs was confirmed by radio-SDS-PAGE under reducing conditions, followed by autoradiographic analysis and Coomassie blue staining. CPM = counts per minute, CPS = counts per second

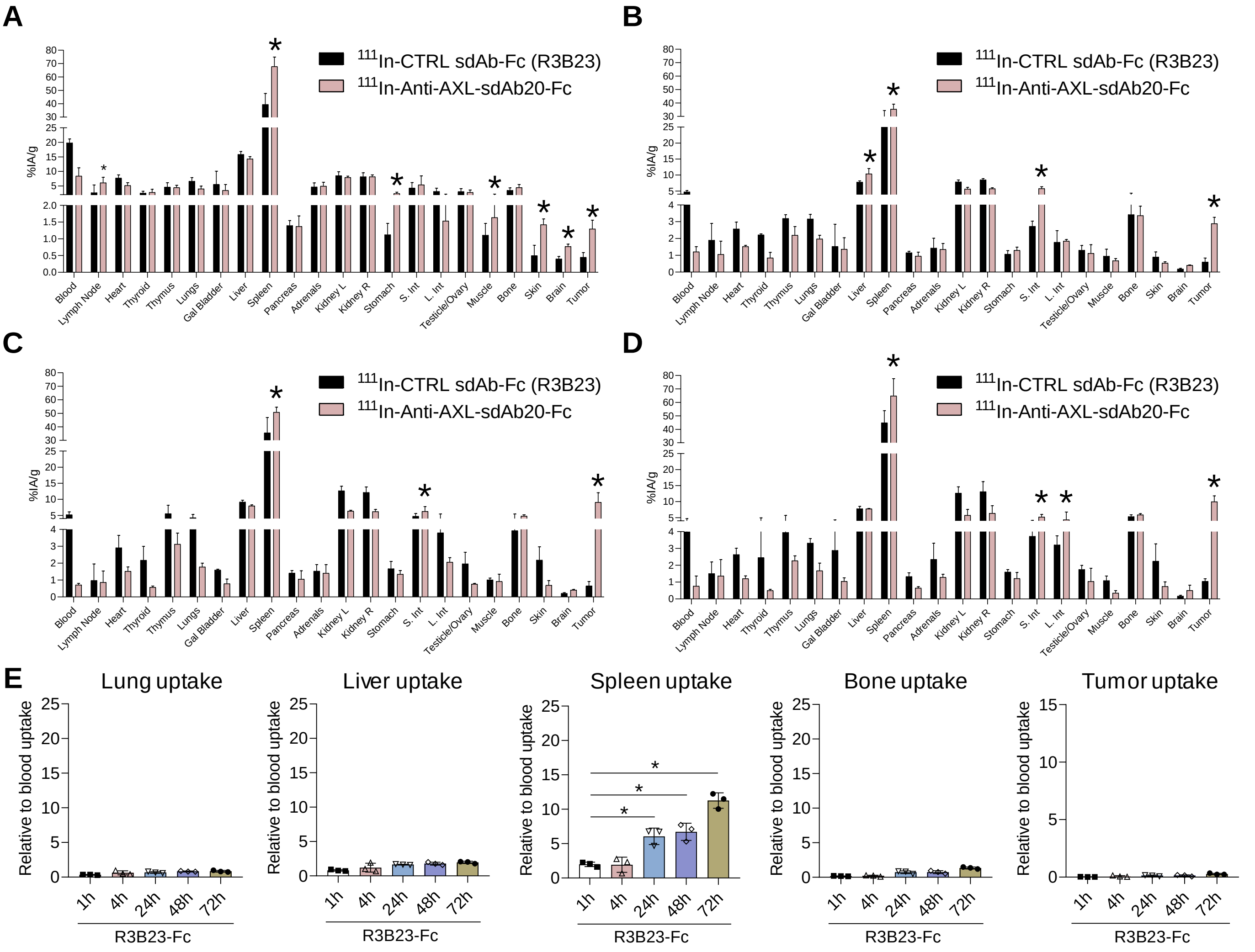

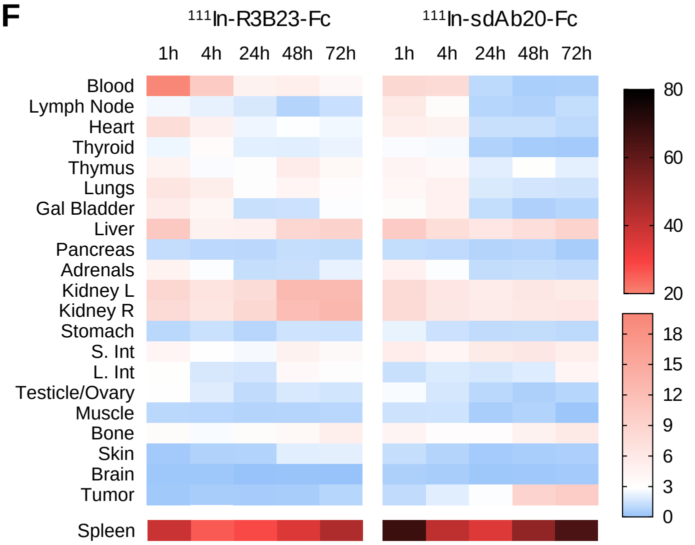

Supplementary Figure 5. (A-D) Graphical representation of the *ex vivo* biodistribution profile of ^{111}In-labeled R3B23-Fc and sdAb20-Fc at 1 h **(A)**, 24 h **(B)**, 48 h **(C)** and 72 h **(D)** post-injection. Results are presented as mean %IA/g ± SD. **(E)** Differences of R3B23-Fc in lung, liver, spleen, bone and tumor, relative to blood uptake. **(F)** Heatmap of accumulation patterns of R3B23-Fc and sdAb20-Fc in various organs at different timepoints. The colour key represents the mean value of tracer accumulation in organs (%IA/g, n=3 for each group). $p \leq 0.05$ (*) was considered statistically significant. IA = injected activity

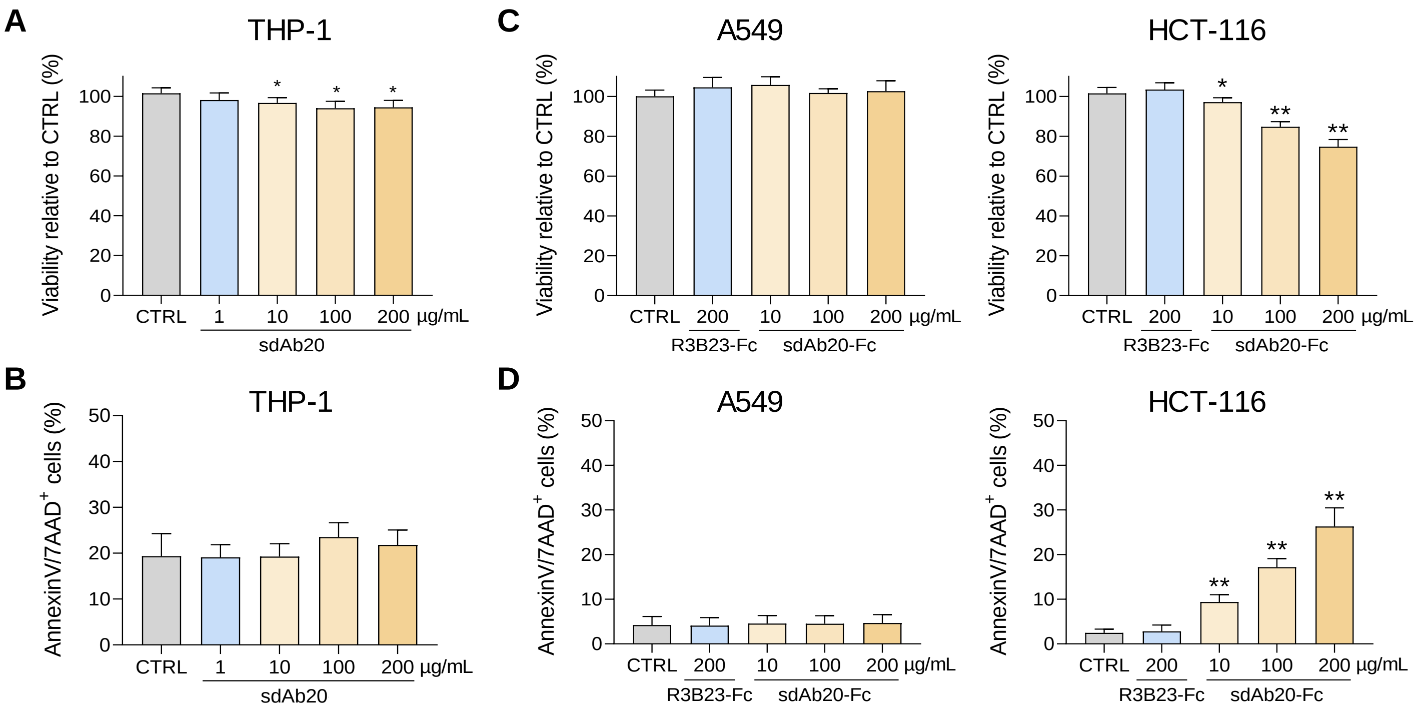

Supplementary Figure 6. (A-B) AML cell line THP-1 was treated with indicated concentrations of sdAb20 (1, 10, 100 and 200 µg/mL) for 72 h. The effect on cell viability (A) and apoptosis (B) was assessed via CellTiter-Glo assay and Annexin V/7-AAD staining, respectively (n=3, ± SD). **(C-D)** A549 (GAS6$^-$/AXL$^+$) and HCT-116 (GAS6$^+$/AXL$^+$) cells were treated for 72 h with R3B23-Fc (200 µg/mL) or sdAb20-Fc (10, 100 and 200 µg/mL) and effects on cell viability and apoptosis were assessed (n=5, ± SD). $p \leq 0.05$ (*) and $p < 0.01$ (**) were considered statistically significant. CTRL = control.

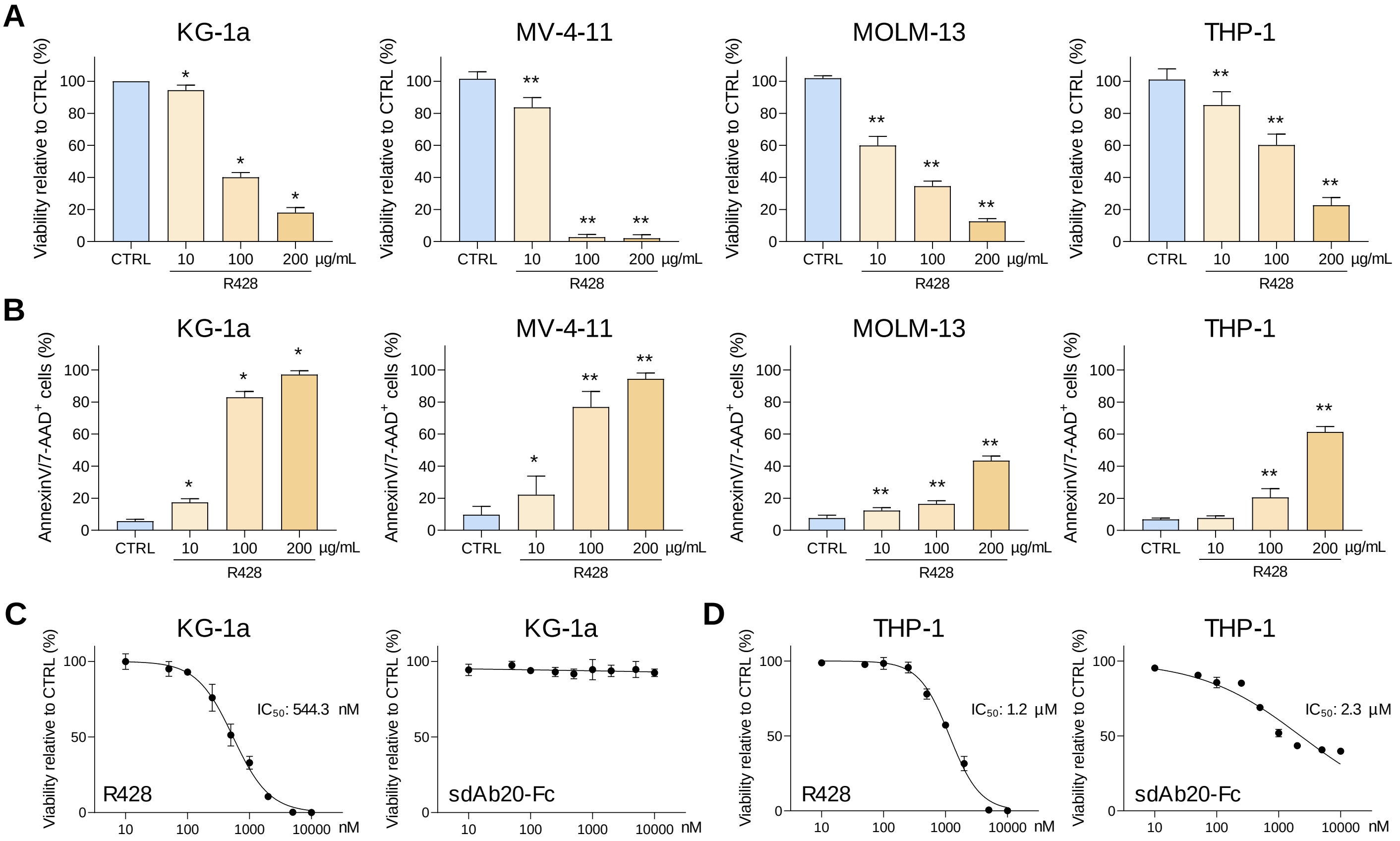

Supplementary Figure 7. (A-B) AML cell lines KG-1a, MV-4-11, MOLM-13 and THP-1 were treated with indicated concentrations of R428 (10, 100 and 200 µg/mL) for 72 h. The effect on cell viability (A) and apoptosis (B) was assessed via CellTiter-Glo assay and AnnexinV/7-AAD staining, respectively (KG-1a: n=6; MV-4-11: n=6; MOLM-13: n=5; THP-1: n=6, ± SD). **(C-D)** AML cell lines KG-1a and THP-1 were treated with increasing molar amounts (10 nM – 10 µM) of R428 (C) and sdAb20-Fc (D) for 72 h. The effect on cell viability was assessed via CellTiter-Glo assay (n=3, ± SD). $p \leq 0.05$ (*) and $p \leq 0.01$ (**) were considered statistically significant. CTRL = control.

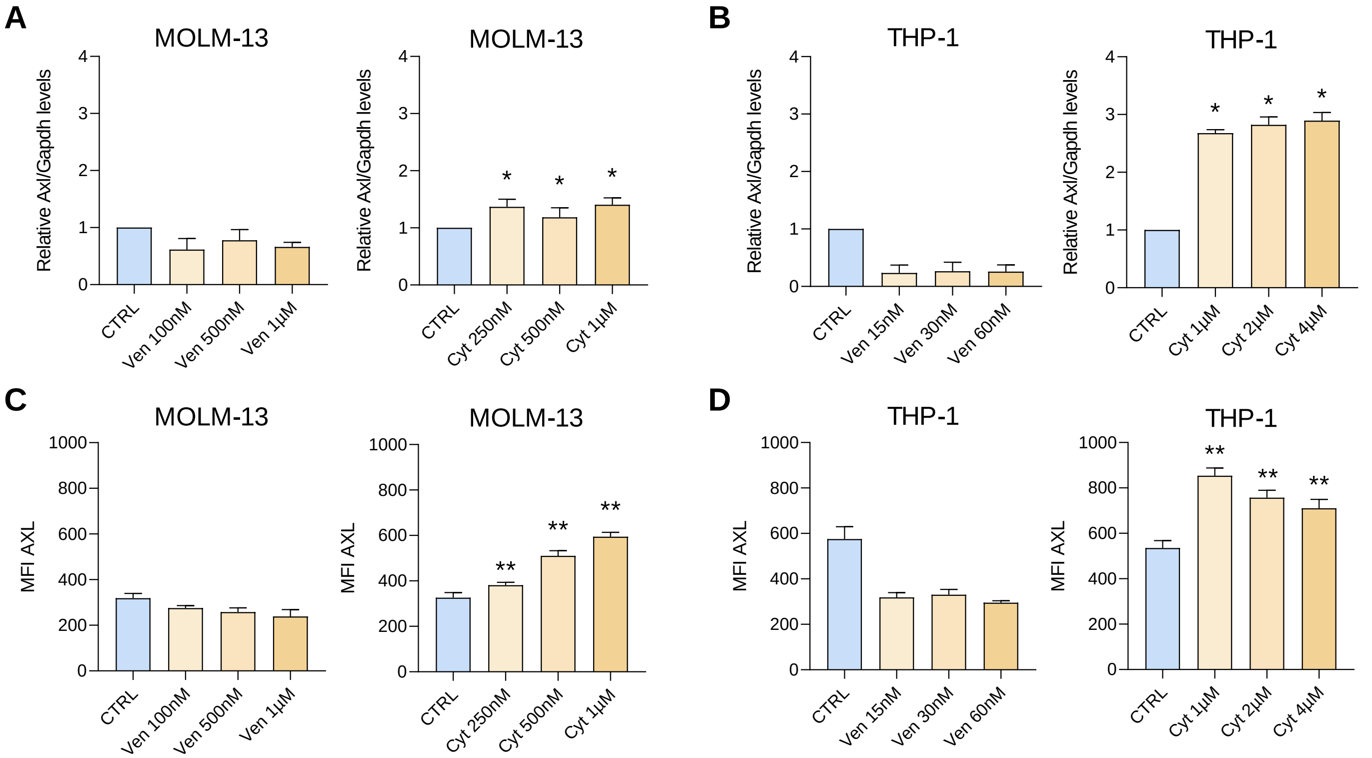

Supplementary Figure 8. (A-B) AML cell lines MOLM-13 (A) and THP-1 (B) were treated with indicated concentrations of venetoclax (Ven) and cytarabine (cyt) for 48 h. Relative *AXL* gene expression was measured by qRT-PCR, the housekeeping gene *GAPDH* was used for data normalization, and differential gene expression was determined using the comparative ΔΔCt method (n=3, ± SD). **(C-D)** Flow cytometric analysis of AXL expression using sdAb20. The irrelevant sdAb R3B23 served as a negative control (n=5, ± SD). $p \leq 0.05$ (*) and $p \leq 0.01$ (**) were considered statistically significant. CTRL = control, MFI = median fluorescence intensity.

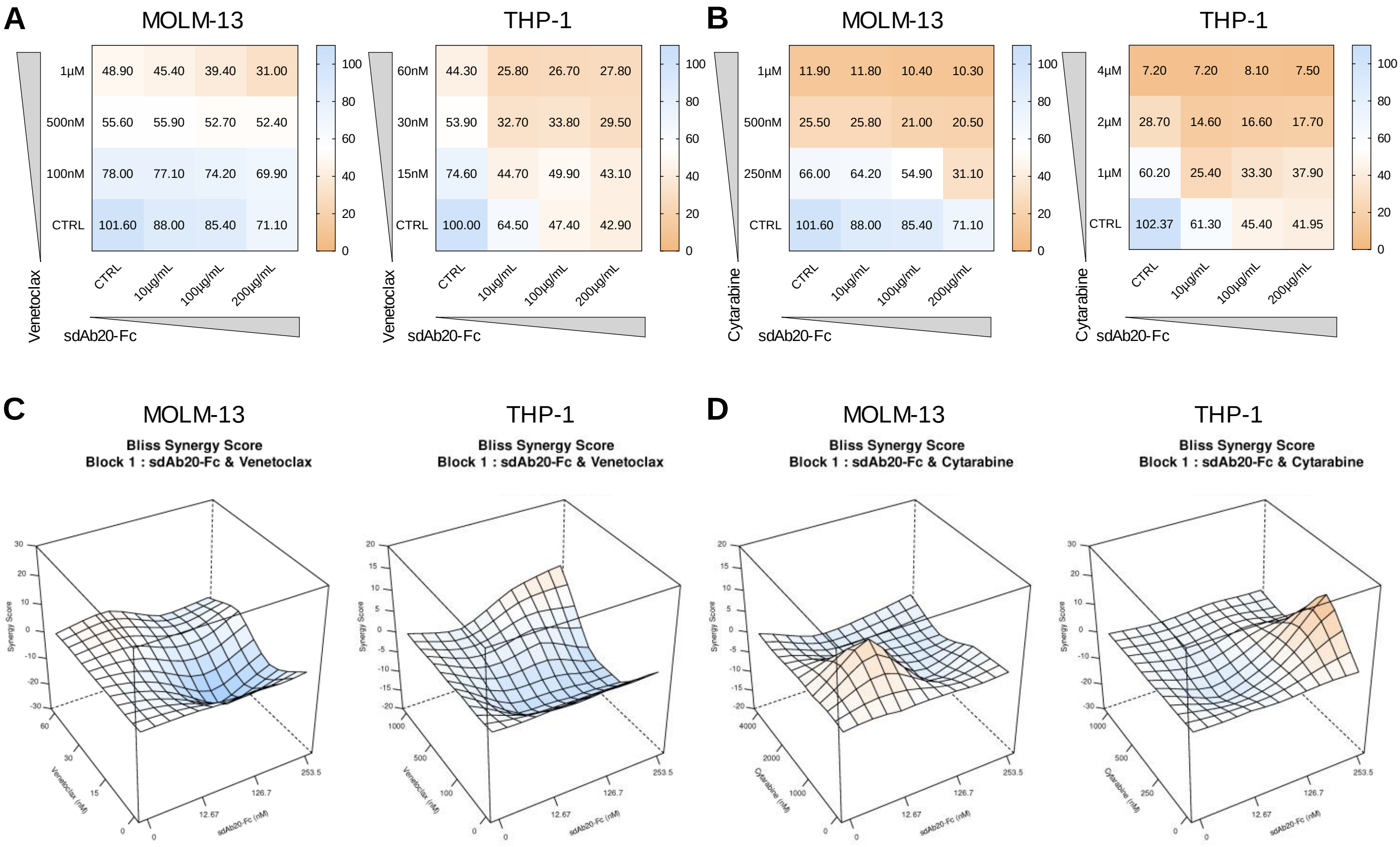

Supplementary Figure 9. (A-B) Heatmaps display the percentage viability for combination treatment of sdAb20-Fc with venetoclax (A) and cytarabine (B). **(C-D)** Synergy of drug interactions was calculated using the BLISS synergy method. Output is generated in 3D format using the SynergyFinder Plus webtool. Data are represented as mean of five samples.

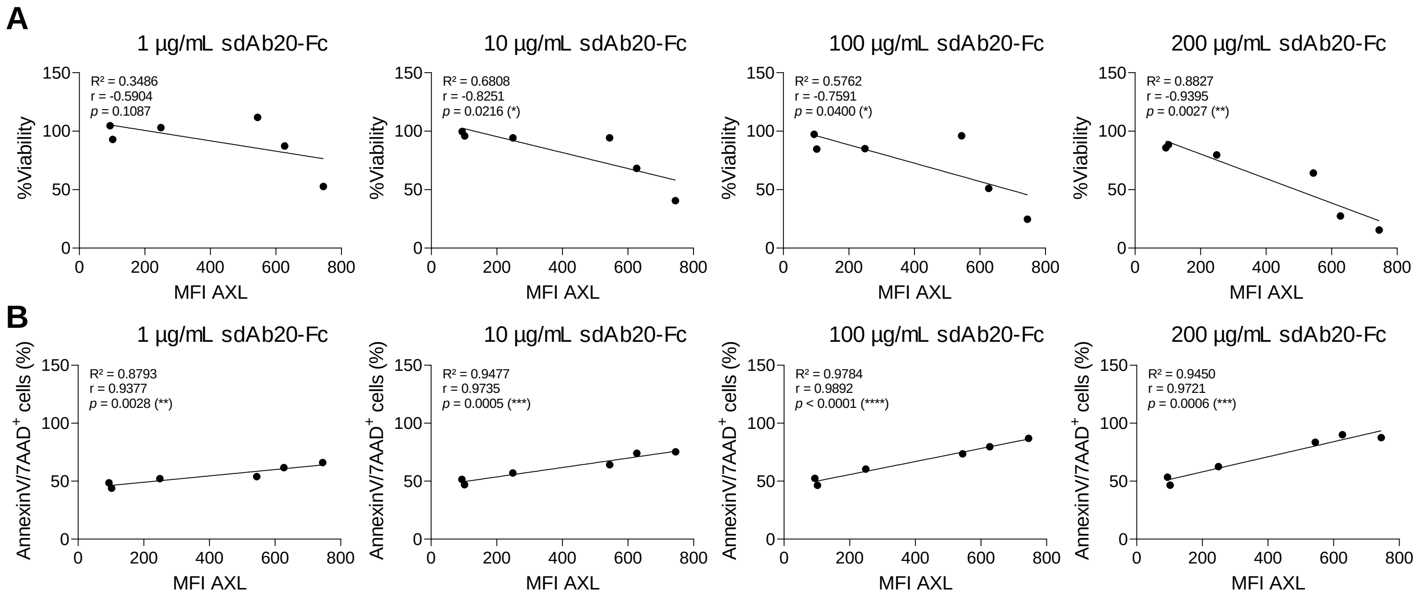

Supplementary Figure 10. (A-B) Analysis of the correlation between AXL expression in blasts of AML patients (x-axis) and the effect of sdAb20-Fc on viability (A) and apoptosis (B)(y-axis). Pearson coefficients (r) and the corresponding coefficients of determination (R^2) and P-values are indicated for each concentration of sdAb20-Fc (n=6). MFI = median fluorescence intensity.
